# Global Nitrogen Deposition Promotes Carbon Sink Formation in Terrestrial Ecosystems

**DOI:** 10.1002/advs.202520069

**Published:** 2026-03-25

**Authors:** Lei Li, Mingyu Xie, Nan Jia, Li‐Dong Mo, Qiang Yu, Fanjiang Zeng, Xiangyi Li

**Affiliations:** ^1^ Key Laboratory of Ecological Safety and Sustainable Development in Arid Lands Xinjiang Institute of Ecology and Geography Chinese Academy of Sciences Urumqi P. R. China; ^2^ Xinjiang Key Laboratory of Desert Plant Roots Ecology and Vegetation Restoration Xinjiang Institute of Ecology and Geography Chinese Academy of Sciences Urumqi P. R. China; ^3^ Cele National Station of Observation and Research for Desert‐Grassland Ecosystems Cele P. R. China; ^4^ University of Chinese Academy of Sciences Beijing P. R. China; ^5^ Department of Plant Biology and Ecology College of Life Science Nankai University Tianjin P. R. China; ^6^ School of Grassland Science Beijing Forestry University Beijing P. R. China

**Keywords:** ^15^N recovery, carbon sink, nitrogen deposition, stoichiometry, terrestrial ecosystem

## Abstract

Nitrogen (N) deposition has been shown to alleviate N limitations on ecosystem productivity, increasing the terrestrial carbon (C) sink. Accurately quantifying the fate of deposited N is essential for reliably estimating its contribution to the global C sink. We conducted an extensive analysis and modeling of 829 observational data points to elucidate the retention patterns of reduced N (NH_x_) and oxidized N (NO_y_) deposited in ecosystems and evaluate their contributions to terrestrial C sinks via stoichiometric scaling. Our results indicate that, on average, 36% of deposited N is retained in terrestrial ecosystems. Globally, the retention of deposited N amounted to 39.15 Tg N yr^−1^, with the retention rates of NH_x_ and NO_y_ being 25.02 and 14.13 Tg N yr^−1^, respectively. Notably, a smaller amount of deposited N absorbed by trees was retained in woody tissues than in nonwoody tissues. Consequently, the estimated N‐induced C sink in terrestrial ecosystems was 0.88 Pg C yr^−1^, accounting for 25.48% of the global terrestrial C sink. This study highlights the global distribution patterns of deposited N retention and the induced biome‐specific C sink, underscoring the importance of incorporating pool‐specific C:N stoichiometry for accurately quantifying ecosystem‐scale carbon sequestration in global modeling.

## Graphical abstract

Nitrogen deposition alleviates ecosystem N limitation and enhances carbon sinks. Using 829 observations, we show 36% of deposited N is retained globally (39.15 Tg N yr^−1^), with distinct NH_x_ and NO_y_ contributions. This retention drives a terrestrial C sink of 0.88 Pg C yr^−1^ (25.48%), highlighting the importance of pool‐specific C:N stoichiometry.

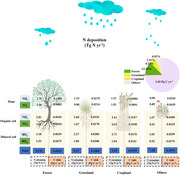

## 1 Introduction

Increased emissions of reactive nitrogen (N) have accelerated the rate of global N deposition [1], enhancing the input of available N to terrestrial ecosystems and consequently amplifying terrestrial ecosystem carbon (C) sinks [2]. The impact of N deposition on terrestrial ecosystem C sinks is contingent upon both the final location of N input to ecosystems and the retention of deposited N in various pools of ecosystems [3]. However, the fate of atmospheric N deposited in the forms of reduced N (NH_x_) and oxidized N (NO_y_) differs substantially, depending on plant uptake preferences, microbe–plant competition for N, and biological N fixation processes [4]. Generally, the preference is higher for ^15^NH_4_^+^ than for ^15^NO_3_^−^ in forests compared to that in other ecosystems [5]. However, a recent synthesis study reported that more ^15^NO_3_^−^ than ^15^NH_4_^+^ was retained more in plants, while the opposite pattern occurred in soils in temperate, subtropical, and tropical forests [6]. In an alpine grassland, it was observed that the recovery rate of ^15^NH_4_^+^ was higher than that of ^15^NO_3_^−^, and most of the N was retained in soil [7]. These contrasting uptake pathways imply different consequences by which deposited N contributes to ecosystem carbon sequestration. Plant N uptake preferences will enhance plant‐mediated C inputs through biomass production and litter return, whereas N retained in soil will be incorporated into organic forms via microbial assimilation and enhanced soil carbon sequestration [8]. In addition, climate change is altering this complex ecosystem through multiple pathways, driven by interacting and often nonlinear mechanisms that influence the fate and cycling of nitrogen [9]. Hence, under climate change scenarios, determining the long‐term dynamics of deposited N and its influence on C sequestration presents a significant challenge.

The magnitude of the N deposition‐induced C sink varies across ecosystem types. In forest ecosystems characterized by a high C/N ratio and long turnover time of woody tissues [10], N deposition stimulates N‐limited trees to store C in woody tissues, where the C is protected from decomposition and respiration‐related release [11], which can greatly increase the C sink of forests. However, in moist tropical forests with fewer N‐limited trees, which are typically characterized by a thin soil organic layer and abundant precipitation, the deposited N is directly transported into the mineral soil layer, increasing the risk of N loss without causing significant changes in the total soil C content [12]. In grassland ecosystems with rapid turnover of plant C, a greater proportion of C is ultimately retained in soil pools through aboveground and belowground litter inputs; therefore, the N deposition‐induced C allocation to plants is relatively lower by proportion [11]. Given the significant heterogeneity of N deposition across ecosystems [13], a spatially explicit investigation of the global pattern for NH_x_ and NO_y_ is a prerequisite for estimating their corresponding contributions to terrestrial C sinks.

Ecosystem C sequestration induced by N retention was quantified via the stoichiometric scaling method [14]. Nadelhoffer et al. [10] conducted ^15^N tracer studies in six European and three North American forest sites and reported that N deposition made little contribution to C sequestration in these temperate forests, which were probably P‐limited areas [15]. Consequently, these ecosystems retained less N and sequestered less C. The impacts of N deposition may thus be weakened due to geographic variations in nutrient limitations, which limit the understanding of the contribution of N deposition to terrestrial C sinks. The previous estimates for N deposition‐induced forest C sinks based on stoichiometry were 0.25–0.38 Pg C yr^−1^ [14, 16, 17], but in these study, fixed empirical values were used for N retention in various forest ecosystems reported in the literature, and forest C sinks were estimated using a fixed C/N ratio without considering the spatial variation in N retention and specific C/N ratios of different plant tissues. Recently, Gurmesa et al. [6] reported that the N deposition‐induced C sink in forests in the 2010s was 21% (0.72 Pg C yr^−1^) of the annual global terrestrial C sink. However, in the study, the authors used the high C/N value of stems to represent the whole‐tree uptake of deposited N and neglected N uptake by nonwoody tree tissues, which may substantially lead to an overestimation of the N deposition‐induced C sink to a large extent [18]. Therefore, a spatially explicit approach that considers various forest types and different parts of the forest is fundamental to assisting our understanding of the nitrogen deposition pathways that affect the carbon sequestration of global forest ecosystems.

Accurate quantification of the fates of NH_x_ and NO_y_ is essential for estimating the impact of N deposition on terrestrial ecosystem C sinks. The global spatial patterns of deposited N and related C sink remain unclear and have rarely been assessed, limiting our understanding of the contribution of N deposition to terrestrial C sinks. We hypothesize the following: (1) The fate of deposited ammonium (NH_4_^+^) and nitrate (NO_3_^−^) differs among ecosystem types, reflecting ecosystem‐specific nitrogen uptake and retention pathways. (2) Ammonium and nitrate deposition lead to different carbon sequestration effects, resulting in contrasting carbon gains per unit nitrogen across ecosystems. Here, we synthesized a total of 829^15^N‐labeled observational data points to (1) investigate the recovery of deposited NH_x_ and NO_y_, (2) reveal the global distribution patterns of retention of deposited NH_x_ and NO_y_ using three N deposition databases, (3) quantify the contributions of N deposition to the global carbon sink through stoichiometric scaling method with corresponding ratios of C/N in the various pools of the ecosystem. This study provides a direct estimate of the total carbon sink by quantifying the nitrogen retention and its ratios of C/N in plants and soil. Our findings reduce the uncertainty in the assessment of N deposition‐related nitrogen‐induced carbon sinks in terrestrial ecosystems and provide insights into carbon sequestration and management under global climate change.

## 2 Results

### 2.1 Recovery of Deposited Nitrogen

**FIGURE 1:**
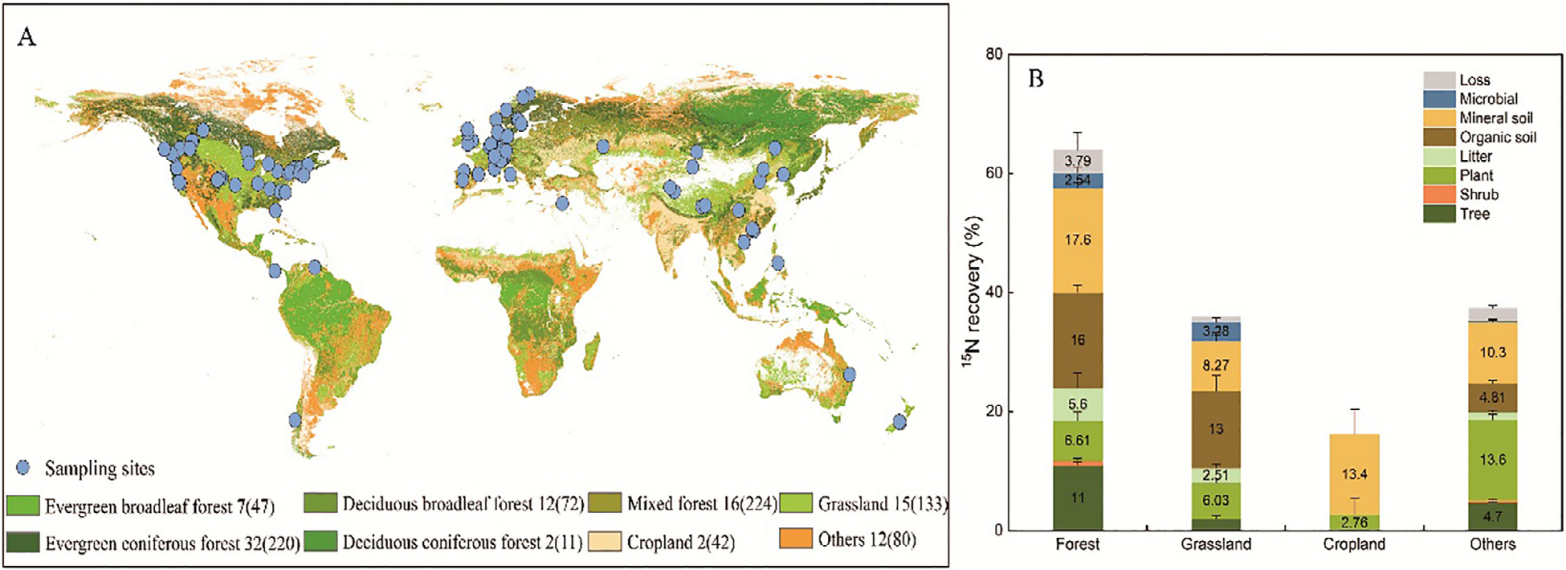
Global distribution map of sampling sites used in this study analysis (A) and the ^15^N recovery of the various pools for different ecosystems (B). In Figure A, numbers corresponding to each ecosystem represent the number of studies, and the number in parentheses represents the number of data observations. Others represent wetland, desert, and tundra. In Figure B, values are presented as mean ± standard error (SE).

To determine the contribution of N deposition to terrestrial C sinks, we first conducted statistical analysis on the recovery of deposited N tracers in various pools in terrestrial ecosystems. Terrestrial ecosystems retain an average of 36% of ^15^N, which is primarily distributed in the organic and mineral soil layers (43%–61% of the total ^15^N recovery) (Table 1). The ^15^N recovery was highest in forests (60%). In contrast, grasslands and other ecosystems showed similar recovery values (35%), and together with croplands (16%), all recovery values were lower than the average (Figure 1 and Table 1).

**TABLE 1:** ^15^N recoveries of ^15^N, ^15^NH_4_^+^, ^15^NO_3_^−^ and ^15^NH_4_^15^NO_3_ in different pools from different ecosystems. The ^15^N recoveries are reported as the means ± SEs. Differences between ecosystems are indicated by different letters (*p* < 0.05).

Ecosystem type	^15^N recovery (%)								
	Tree	Shrub	Grass	Litter	Organic soil	Mineral soil	Microbial biomass	Loss	Total (excluding loss)
Global ecosystem	10.32 ± 0.58	2.08 ± 0.33	17.45 ± 1.24	20.13 ± 1.78	27.24 ± 1.04	22.91 ± 0.95	7.27 ± 0.69	10.02 ± 1.47	107.40 ± 1.06
Forest	11.01 ± 0.60	0.81 ± 0.32	6.61 ± 1.46b	5.60 ± 2.45a	16.00 ± 1.17	17.58 ± 0.92a	2.54 ± 0.94	3.79 ± 2.92	60.15 ± 1.38a
Grassland	2.05 ± 0.52	∖	6.03 ± 0.64b	2.51 ± 0.61b	12.99 ± 2.40	8.27 ± 1.45c	3.28 ± 0.64	0.78	35.13 ± 2.48b
Cropland	∖	∖	2.76 ± 2.77b	∖	∖	13.37 ± 4.19ab	∖	∖	16.13 ± 3.35c
Others	4.70 ± 0.08	0.43 ± 0.04	13.59 ± 0.78a	1.27 ± 0.29b	4.81 ± 0.49	10.31 ± 0.46bc	0.17 ± 0.07	2.09 ± 0.48	35.28 ± 1.98b
^15^NH_4_^+^									
Forest	11.63 ± 1.61	0.97 ± 0.52	4.93 ± 2.59b	7.97 ± 1.28a	24.73 ± 4.90a	23.99 ± 4.12a	4.39 ± 1.34	3.32 ± 1.38	78.61 ± 1.98a
Grassland	2.44 ± 0.91	∖	7.99 ± 0.84ab	2.74 ± 0.94ab	32.95 ± 6.06a	15.77 ± 2.91ab	7.35 ± 1.06	0.20	69.24 ± 2.94a
Cropland	∖	∖	8.27 ± 2.77a	∖	∖	28.61 ± 6.90a	∖	∖	36.88 ± 4.87b
Others	13.40	∖	1.54 ± 0.56b	0.60 ± 0.22b	2.35 ± 1.13b	8.99 ± 0.40b	∖	0.11 ± 0.03	26.88 ± 1.69b
^15^NO_3_^−^									
Forest	11.49 ± 2.35	1.46 ± 0.59	6.30 ± 3.16	6.14 ± 2.23	15.72 ± 3.49	17.46 ± 4.33a	1.95 ± 0.39	8.05 ± 3.14	60.52 ± 3.04a
Grassland	3.71 ± 0.65	∖	6.74 ± 0.69	4.31 ± 0.77	6.03 ± 1.14	4.22 ± 0.49b	0.39 ± 0.09	2.13	25.40 ± 2.95b
Cropland	∖	∖	∖	∖	∖	11.51 ± 1.76b	∖	∖	11.51 ± 1.76c
Others	∖	∖	6.88 ± 2.12	2.34 ± 0.86	2.45 ± 0.92	5.79 ± 0.11b	∖	0.12 ± 0.02	17.46 ± 3.40b
^15^NH_4_^15^NO_3_									
Forest	9.92 ± 2.13	∖	8.60 ± 1.43b	2.69 ± 0.83a	7.54 ± 1.54	11.29 ± 1.79ab	1.29 ± 0.54	∖	41.33 ± 2.30a
Grassland	∖	∖	3.34 ± 0.40b	0.49 ± 0.11b	∖	4.83 ± 0.95b	2.09 ± 0.76	∖	10.75 ± 2.21b
Cropland	∖	∖	∖	∖	∖	∖	∖	∖	∖
Others	0.70 ± 0.26	1.28 ± 0.12	32.35 ± 1.31a	0.87 ± 0.33b	9.63 ± 0.81	16.15 ± 0.52a	0.50 ± 0.19	6.04 ± 1.44	61.48 ± 3.31a

The recoveries of ^15^NH_4_^+^ and ^15^NO_3_^−^ from plant biomass (18% and 19%, respectively) were significantly lower than those from soil (49% and 33%, respectively) in forests (Table S2). Notably, the nonwoody components retained an average of 14% ^15^N, approximately twice that of woody tissues (7.52%) (Table S3). In addition, the N retained by forest litter accounted for 17%–30% of the total N retained, which may increase N retention in the soil organic layer (Table S2). In grassland ecosystems, a greater proportion of ^15^NH_4_^+^ was retained in soil, and only small fractions were taken up by plants, whereas the retention of ^15^NO_3_^−^ in plants and soil was comparable (Table 1). Although the total recovery of ^15^NH_4_^+^ in all ecosystems was greater than that of ^15^NO_3_^−^ (Table 1), the ^15^N paired‐isotope experiments (simultaneous labeling of NH_4_^+^ and NO_3_^−^) indicated that more ^15^NO_3_^−^ was retained by plants and mineral soils (Figure S1B; Figure 1D).

The recovery of deposited N differed among forest components and among different types of forests. In most forest types, plants retained more ^15^NO_3_^−^, except in evergreen coniferous forests and mixed forests, where the retention rates of ^15^NH_4_^+^ and ^15^NO_3_^−^ were similar (Table S2). In addition to deciduous coniferous forest and mixed forest mineral soil, forest organic soil and mineral soil preferentially retain ^15^NH_4_^+^ (Table S2). We found that the average total ecosystem ^15^N recovery decreased from 73% for < 100 days to 57% for 500–1000 days (Figure S2). While the total ^15^N average recovery of the ecosystem tended to decrease over time, the total ^15^N recovery was sometimes >100% due to the use of non‐mutually exclusive categories, such as microbial biomass and soil pools (Table S2 and Figure S2).

### 2.2 Global Patterns of Deposition and Retention of NH_x_ and NO_y_

**FIGURE 2:**
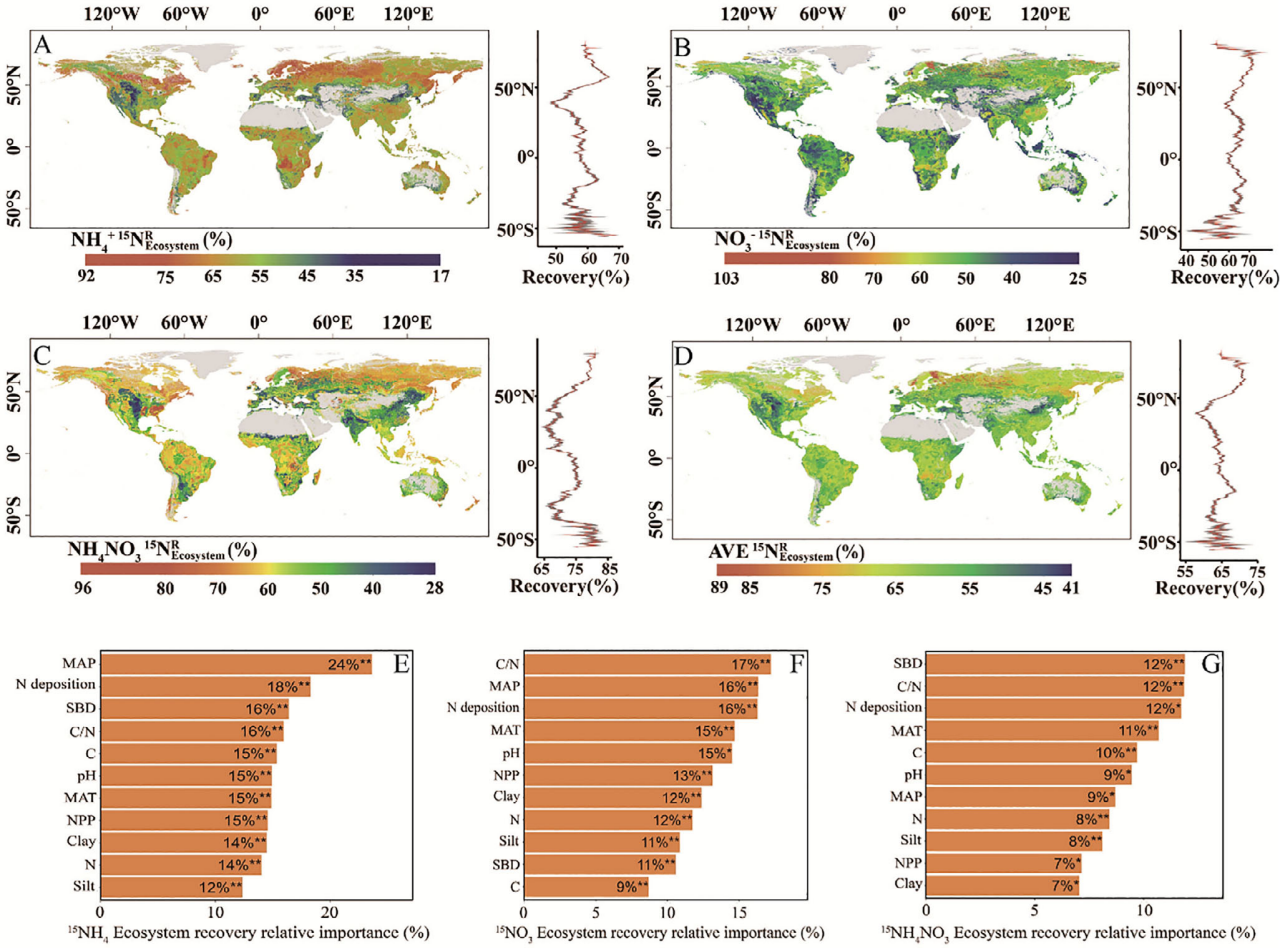
Modeled global patterns of ecosystem retention of ^15^NH_4_^+^ (A), ^15^NO_3_^−^ (B), ^15^NH_4_^15^NO_3_ (C), their average values (D), and their relative importance (E‐F) in terrestrial ecosystems. The values were predicted using a data‐driven random forest model with global climate, vegetation, and soil properties. The grey shadows represent the fifth and 95th quantiles, which represent the 95% prediction interval of the random forest model. In figures E–G, ^*^ indicates the significance of each explanatory variable in the model (^*^ *p* < 0.05 ^**^ *p* < 0.01).

Globally, all three ^15^N isotope tracers exhibited high recoveries in plants from the northern region (Figure S4), with ^15^NH_4_^+^ having significantly greater recovery than ^15^NO_3_^−^ in organic soils from the northern and temperate regions, whereas ^15^NH_4_^15^NO_3_ had greater recovery in both organic and mineral soils from the subtropical and tropical regions (Figure 2; Figures S5 and S6 and Table S4).

Our modeling results showed the amount of deposited N retained was 39.15 Tg N yr^−1^, which included NH_x_ and NO_y_, which accounted for 25.02 and 14.13 Tg N yr^−1,^ respectively. Specifically, the retention rates of NH_x_ in plant, organic soil, and mineral soil were 5.10, 9.75, and 10.17 Tg N yr^−1^, respectively, whereas those of NO_y_ were 4.72, 4.52, and 4.89 Tg N yr^−1^, respectively. Forest ecosystems retained 7.75 Tg N yr^−1^ of NH_x_, which was greater than the NO_y_ value of 5.50 Tg N yr^−1^, and the retention of NH_x_ was also greater than that of NO_y_ in grasslands, croplands, and other ecosystems (Table S5). In forest ecosystems, the amount of N retained in nonwoody tissues, such as foliage, fine roots, and bark, was 2.41 Tg N yr^−1^ greater than that in woody tissues, such as stems, coarse roots, and branches, with N retention of 1.75 Tg N yr^−1^ (Table S6).

### 2.3 Spatial Patterns of the Global Carbon Sink

**FIGURE 3:**
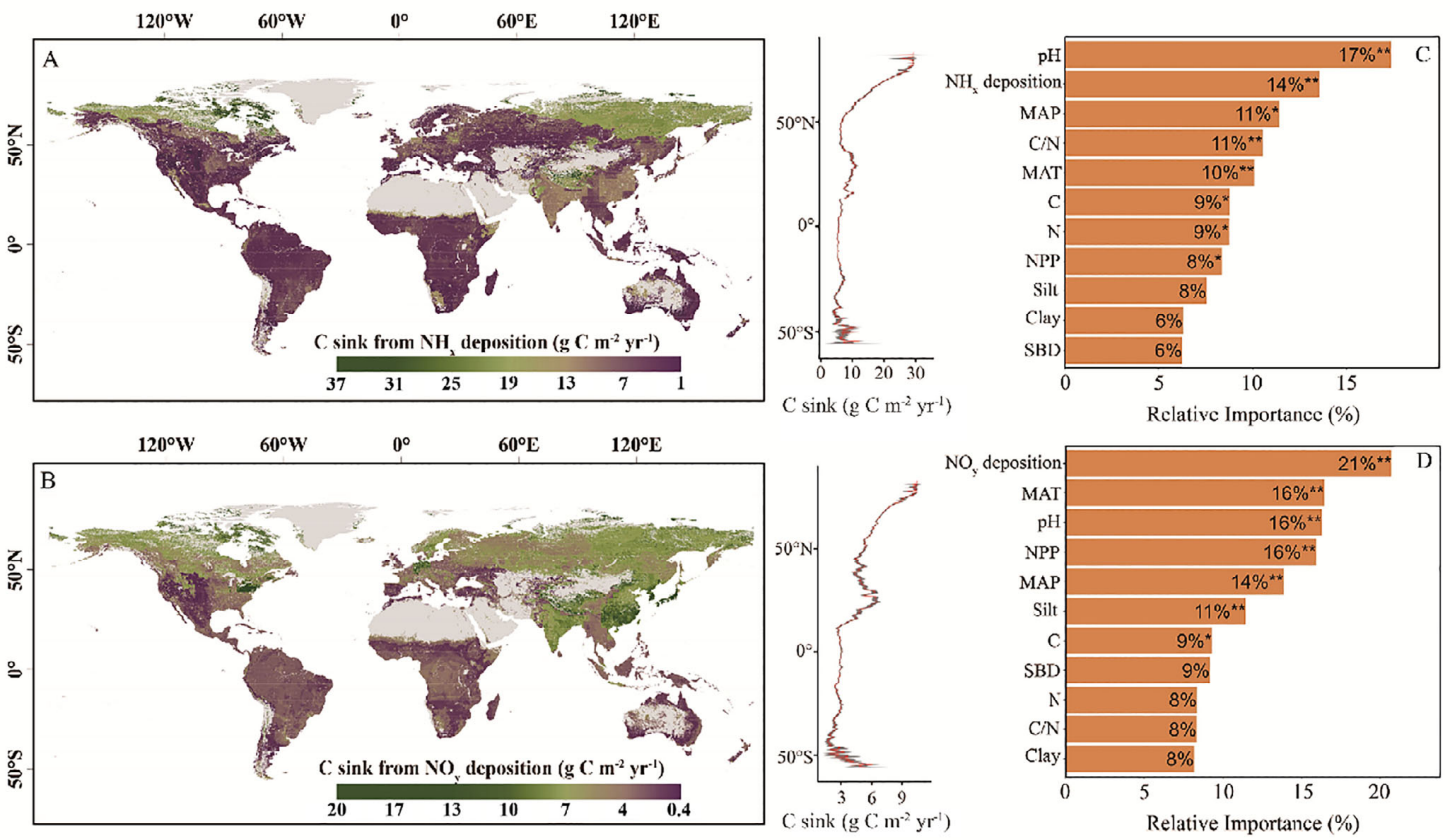
Modelled global patterns and relative uncertainty of global terrestrial C sink induced by NH_x_ (A), NO_y_ (B), and their relative importance (C, D). Values were predicted using a data‐driven random forest model with global climate, vegetation, and soil properties. The grey shadows represent the fifth and 95th quantiles, representing the 95% prediction interval of the random forest model. In figures C and D, ^*^ indicates the significance of each explanatory variable in the model (^*^ *p* < 0.05, ^**^ *p* < 0.01).

The N‐induced terrestrial C sinks in the northern region and some subtropical and tropical regions were significant (Figure S9; Figure 3). From 2010 to 2014, the model simulation showed that the total inorganic N deposited was 55.27 Tg N yr^−1^, and the N‐induced C sink was 0.88 Pg C yr^−1^ (Table 2; Table S7), which accounted for approximately 25.48% of the total terrestrial C sink, according to the global carbon budget [19]. Specifically, the N‐induced C sink in forests was 0.45 Pg C yr^−1^, accounting for 51.51% and 13.13% of the total terrestrial N‐induced C sink and total global C sink, respectively. The contribution of NH_x_ deposition to the forest C sink was 0.28 Pg C yr^−1^, which was greater than the NO_y_ deposition of 0.18 Pg C yr^−1^. A large part of the N‐induced forest C sink was derived from plants due to the high C/N ratio of forest wood, which accounted for 52.22% of the total forest C sink. The N‐induced C sinks in grasslands, croplands, and other ecosystems were 0.15, 0.07, and 0.21 Pg C yr^−1^, respectively (Table 2; Figure S9 and Table S9).

**TABLE 2:** Contribution of deposited NH_x_ and NO_y_ to the C sink in plants, organic soil, and mineral soil across ecosystems. The numbers in parentheses represent 95% confidence intervals.

Ecosystem type	Area (million km^2^)	C sink (Pg C yr^−1^)		
		NH_x_ deposition	NO_y_ deposition	Total N (NH_x_+NO_y_) deposition
Deciduous broadleaf forest	12.12	0.0358 (0.0302, 0.0981)	0.0292 (0.0144, 0.0726)	0.0650 (0.0446, 0.1707)
Evergreen broadleaf forest	12.38	0.0399 (0.0342, 0.0917)	0.0339 (0.0192, 0.0483)	0.0738 (0.0534, 0.1400)
Deciduous coniferous forest	10.13	0.1338 (0.0389, 0.1549)	0.0616 (0.0354, 0.0715)	0.1954 (0.0743, 0.2164)
Evergreen coniferous forest	14.50	0.0616 (0.0299, 0.1979)	0.0442 (0.0234, 0.0843)	0.1058 (0.0533, 0.2822)
Mixed forest	3.41	0.0067 (0.0061, 0.0353)	0.0062 (0.0051, 0.0189)	0.0129 (0.0112, 0.0542)
Forest	52.54	0.2778 (0.1393, 0.5770)	0.1751 (0.0976, 0.2856)	0.4529 (0.2368, 0.8635)
Grassland	26.47	0.0859 (0.0469, 0.3738)	0.0601 (0.0229, 0.1695)	0.1460 (0.0698, 0.5433)
Cropland	12.23	0.0427 (0.0268, 0.1116)	0.0301 (0.0125, 0.0743)	0.0728 (0.0393, 0.1859)
Others	37.44	0.1125 (0.0740, 0.7409)	0.0950 (0.0331, 0.2375)	0.2075 (0.1071, 0.9784)
Ecosystem	128.68	0.5189 (0.2869, 1.8042)	0.3603 (0.1660, 0.7670)	0.8792 (0.4530, 2.5712)

Notably, N deposition was positively correlated with the N‐induced C sink (Figure S11). The carbon gain per unit of N deposited in global terrestrial ecosystems was 15.91 kg C kg^−1^ N, with the carbon gain of NO_y_ deposition (17.97 kg C kg^−1^ N) being greater than that of NH_x_ deposition (14.73 kg C kg^−1^ N). The carbon gain per unit of N deposited in forests was highest at 25.40 kg C kg^−1^ N but was lowest in croplands at 5.40 kg C kg^−1^ N (Tables S11 and S12).

## 3 Discussion

### 3.1 The Fate and Variations in Deposited Nitrogen

Global ^15^N tracer experiments revealed that 21% of the deposited N infiltrated soil and was subsequently fixed, 12% was absorbed by plants, and only 3% was retained in litter (Table 1). It seems plausible that a significant amount of deposited N may have been lost as gas and through leaching, but this fraction accounted for merely 2%–6% of our observed data. Only a few articles among our collected studies reported N losses in the gaseous form and/or via leaching. In addition, the ^15^N added in some experiments was rapidly lost at the beginning of the experiment, possibly due to the physical absorption of ^15^N on litter and mineral soil surfaces and/or through abiotic processes such as leaching or erosion caused by heavy rain [12, 20]. Our findings indicate that woody tissues are not the primary sink for N deposition in trees, but most ^15^N tracers were retained in nonwoody tissues, such as foliage, fine roots, and bark (Table S3).

Our results suggest that trees preferentially take up NO_3_^−^ in forest ecosystems, whereas more NH_4_^+^ is retained in mineral soils (Table 1; Table S2). However, the ^15^NO_3_^−^ retained in plants and mineral soils was greater than the ^15^NH_4_^+^ retained according to ^15^N pairing experiments (Figure S1). This may be due to the preferential assimilation and retention of ^15^NH_4_^+^ via abiotic mechanisms such as microbial and soil sorption, which leads to the accumulation of ^15^NH_4_^+^ on soil particles [21]. In contrast, ^15^NO_3_^−^ moves more readily to the root surface through diffusion and mass flow than ^15^NH_4_^+^ does [22], and ^15^NO_3_^−^ uptake allows plants to minimize competition with soil microbes for ^15^NH_4_^+^ [23]. In addition, we found that more ^15^N was retained in soil due to the positive buffering effect of the litter layer on N deposition in forest ecosystems, which could transform the litter layer from a N sink to a source that gradually releases N to the soil [24]. The organic layer and litter layer of forest ecosystems were initially the main ^15^N sinks (Figure S2) [25], and this variation in recovery could explain a part of the change in total ^15^N retention (Figure S12A,B). With time, there were also cases in which the ecosystem recovery in one time range was greater than that in the previous time range. This may have occurred because multiple additions of ^15^N tracers were carried out in some studies, which limited our ability to determine the change in ^15^N recovery over time.

### 3.2 Global Nitrogen Retention

The global inorganic N deposition data from 2010 to 2014 indicated that the NH_x_ retention (25.02 Tg N yr^−1^) was greater than the NO_y_ retention (14.13 Tg N yr^−1^) (Table S5). Our findings suggest that the retention of N in soil is 3 times greater than that in plants and that more NO_3_^−^‐N leaching and N gas escape may occur when more deposited N is fixed in the soil [10, 25]. Within forests, deposited N was more likely to be absorbed by fine roots, foliage, and other parts of trees, whereas the retention of deposited N in stems with long turnover time was small (Table S6). Broadleaf forests retained more ^15^NO_3_^−^ than coniferous forests, suggesting that broadleaf trees preferentially absorb NO_3_^−^‐N rather than NH_4_^+^‐N [26]. In addition, the retention of deposited N was regulated by the soil available nutrient content, which is associated with the status of N limitation [6, 25]; deciduous coniferous forests, which are highly restricted by N in the northern region, retained more than 80% of the deposited N (Table S5), and ^15^N recovery was negatively correlated with the mineral soil N content (Figure S12C,D). However, our study revealed that the N retention rate of evergreen broadleaf forests and deciduous broadleaf forests with relatively high N contents was 70% (Table S5). This may have occurred because C─N covalent bonds exist in litter and soil organic matter, which can slow the N cycle, thereby increasing N retention as soil C increases [27], suggesting a nonnegligible N retention capacity even in ecosystems with high N availability.

**FIGURE 4:**
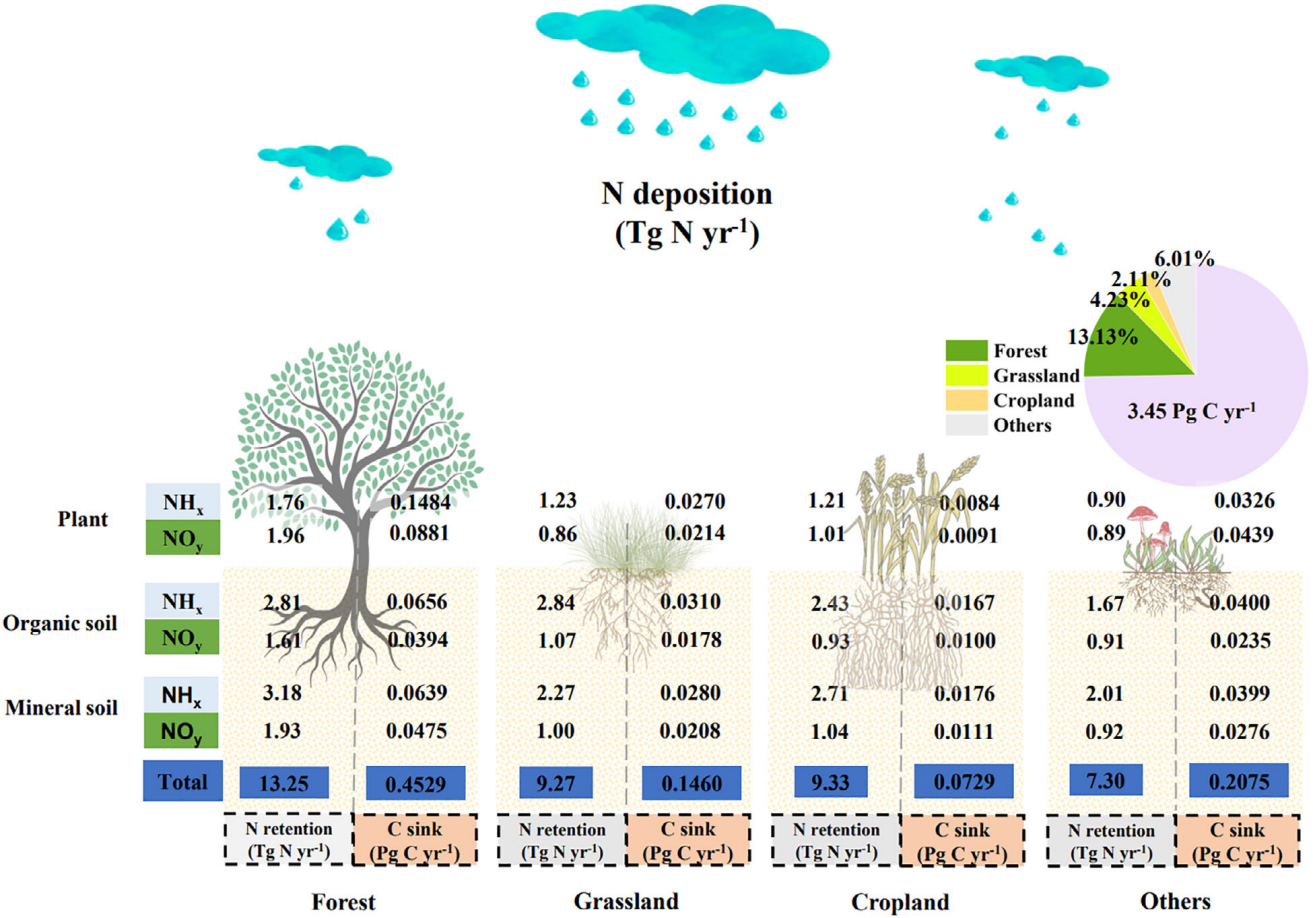
Schematic diagram of N retention of atmospheric inorganic N deposition in plant and soil pools and related C sink induced from 2010 to 2014. Schematic representation of N retention (left) and N‐induced C sink (right) in different ecosystems. The pie chart shows the N‐induced C sink in ecosystems as a proportion of the global terrestrial C sink (2021). Others represent wetland, desert, and tundra.

Previous research has demonstrated that forests are N‐limited in high‐latitude regions; consequently, deposited N is predominantly retained within plant and organic soils. Conversely, N‐rich forests in low‐latitude regions tend to retain the N deposited in mineral soils [6]. This finding is consistent with our observations (Figure 4; Figures S4–S6) and is attributed mainly to limited precipitation in northern N‐limited forests, coupled with a thicker soil organic layer that obstructs the transport of N to the mineral layer [28]. In contrast, less developed organic layers and rapid litter turnover at lower latitudes could facilitate the direct transfer of deposited N into the mineral soil layer [29], potentially contributing to the low capacity of the organic soils in these forests to retain deposited N.

### 3.3 Global Nitrogen Deposition‐Induced Carbon Sink

We estimated the C sink due to N deposition by stoichiometric scaling, and the results indicated a widespread stimulatory effect of nitrogen input on ecosystem carbon retention (Figure S9). Specifically, the N‐induced terrestrial C sink decreased from cold to tropical regions. The low temperatures prevalent in northern regions restrict N mineralization, whereas the higher temperatures in tropical areas intensify N leaching and denitrification [30, 31].

We predicted an N deposition‐induced terrestrial C sink of 0.88 Pg C yr^−1^, which is 0.86–3.38 times greater than that reported in some previous studies (Table S12) [32, 33, 34]. Our findings suggest that, although tree woody tissue is the smallest N sink (Table S6), it is the greatest C sink due to its high C/N ratio and extended turnover time [10]. It is worth noting that soil carbon sequestration was primarily derived from plant and microbial necromass rather than directly driven by nitrogen deposition [35]. Consequently, over short time scales,^15^N tracer experiments may not adequately capture changes in soil carbon stabilization because the process of litter decomposition is relatively slow [36, 37], while these experiments may still offer a valuable perspective on carbon stabilization. In addition, we performed sensitivity tests using the lower bounds of the soil C/N ratio to investigate the influences on our estimates (Table S10). Previous estimates of the contribution of N deposition to the forest C sink based on stoichiometry were in the range of 0.25–0.376 Pg C yr^−1^ [14, 16, 17], which are similar to our estimates (Table S12). However, in the previous studies, researchers assumed that the N retention rates and C/N ratios were fixed, overlooking differences among ecosystems, which may lead to uncertainty in the estimated values. While a recent study addressed the above issues [6], it failed to distinguish N uptake between woody and nonwoody tissues of trees, and because a higher C/N ratio was used for woody tissue (4–10 times greater than that of nonwoody tissue), it significantly overestimated the C sink induced by N deposition. Although several studies have estimated the C sink through the synthesis of published data from N addition experiments in other ecosystems [38, 39], the levels of N addition were high in these studies, far exceeding those associated with atmospheric N deposition. Instead, the ^1^ ^5^N addition rates in our dataset averaged approximately 40% of ambient atmospheric nitrogen deposition, making these rates unlikely to substantially alter the underlying dynamics of the ecosystem nitrogen cycle. Therefore, they may not fully capture the effects of N retention changes on ecosystem C sinks under more natural N deposition conditions.

Although our study revealed greater retention and induced carbon sinks for NH_x_ in the ecosystems (Table 2; Table S5), greater carbon gain occurred from NO_y_ deposition than from NH_x_ deposition due to the greater uptake of ^15^NO_3_^−^ by plants and due to the significantly greater C/N ratio observed in plants than in soils. In relatively nitrogen‐rich ecosystems, nitrate‐dominated NO_y_ inputs may enhance soil carbon sequestration primarily by suppressing soil CO_2_ emissions and reducing dissolved organic carbon losses, rather than by stimulating plant productivity [40, 41, 42]. By contrast, NH_x_ inputs are more likely to influence carbon cycling indirectly through plant‐mediated pathways by enhancing primary productivity in nitrogen‐limited systems, although associated soil acidification may offset soil carbon gains [40, 41, 42, 43]. Consequently, NO_y_ deposition plays a more substantial role in contributing to the global C sink.

### 3.4 Implications and Uncertainties

Forests contribute the most to the C sink, particularly in N‐limited regions at high latitudes [6], and we suspect that afforestation under suitable climatic conditions effectively increases the C sink in response to N deposition. Tropical and subtropical forests play pivotal roles in limiting global warming and alleviating climate change [44], and N inputs can reduce CO_2_ fluxes and increase soil carbon sequestration in mature tropical forests [42]. Therefore, future research should be focused on N‐rich regions to reveal potential C sinks under N deposition to improve the predictability of C sinks under global changes under N deposition. Considering the difference in the C gain per unit of N deposited between NH_x_ and NO_y_, increased NO_y_ fertilization may effectively promote C accumulation in grasslands, croplands, and other ecosystems. Our study demonstrated the operability and robustness of the stoichiometric scaling method in the estimation of the deposited N‐induced C sink. First, the C sink is estimated by a simple mathematical formula, and the need for a complicated and multivariable carbon model is eliminated. Second, the data that were used were collected from experiments involving small amounts of ^15^N tracer addition, which accounted for natural N deposition without causing disturbances to the intrinsic carbon and nitrogen cycles. Third, the method could be used to accurately quantify the carbon sink through highly accessible nitrogen retention and C/N ratios. Nevertheless, this approach has several limitations in that it assumes that the plant and soil C:N ratios are fixed, even though the C:N ratios decrease under nitrogen deposition conditions [45]. The estimates are based on fixed N retention and distribution over time and ignore the dynamics of N retention and distribution during the plant growing season and the turnover of C and N. Moreover, this is an indirect method of measurement that does not include actual direct measurements of the C sink. Although numerous studies have indicated that N application can reduce soil CO_2_ emissions [46], increased N availability might also accelerate soil C loss [47]. Notably, these results are derived from excess N addition experiments rather than from natural deposition conditions and suggest that further research is needed to quantify soil carbon sequestration and mineralization under N deposition using isotopes. These limitations highlight the need for additional measurements of deposited N retention and its effects on soil carbon sequestration and mineralization, as it is essential for accurately determining the net C sink capacity of ecosystems under N deposition.

Our global estimates are subject to some uncertainties. First, ^15^N‐labeling experiments were conducted in East Asia, North America, and Europe, and sparse data were collected in regions such as Africa, South America, and Oceania (Figure 1). This uneven geographical distribution is a critical source of uncertainty. It is particularly concerning because recent studies show that global N deposition hotspots are shifting toward developing countries in mid‐ and low‐latitude regions [48], which are precisely the areas underrepresented in our dataset. This substantial data gap may limit the accuracy of global carbon sink estimates, and more extensive and direct measurements in these regions are needed in the future to improve precision. Second, despite our meta‐analysis not detecting a significant overall relationship between N recovery and labeling time (Figure S8), we acknowledge the importance of the duration of ^15^N labeling. Additionally, a recent decade‐long ^1^ ^5^N labeling experiment demonstrated that recovery can strongly depend on and decrease with experimental duration, suggesting the existence of saturation thresholds [8]. Accordingly, the carbon sequestration estimated here should be interpreted as a duration‐inclusive mean response based on existing tracer observations, rather than a long‐term equilibrium maximum. Over a longer timescale, cumulative N deposition may decrease the N retention and C/N ratio of ecosystems and increase N loss caused by leaching/runoff and denitrification [14]. Moreover, the net carbon sink of long‐term N deposition may differ from this estimate, due to the changes in soil microbial activity [40], greenhouse gas emissions (such as N_2_O) [41], and other indirect processes [8]. These duration‐related limitations of the experiment may increase the uncertainties in estimating N‐induced C sequestration. Finally, some studies reported only the recoveries of a few pools, not the total ecosystem recovery (Supplementary Dataset), which resulted in increased uncertainty in N retention.

Despite the stabilization expected in the future atmospheric N deposition flux [17] and the decrease expected in the proportion of deposited NO_y,_ which has a relatively high unit C gain [49, 50], we observed a positive correlation between N deposition and the related C sink induced (Figure S11), and our study implied that the impacts of N deposition on the terrestrial C sink would decrease in the future. In addition, under large‐scale disturbances caused by human activities, such as deforestation and wildfires, the C sink is likely to be overestimated [51]. This occurs not only because of a reduction in biomass but also because of lower N recoveries under reforestation or natural recovery conditions [28, 52, 53]. Thus, our estimate is likely to be an upper limit of the global forest C sink induced by N deposition.

## 4 Conclusions

This study estimates the C sinks induced by N deposition in terrestrial ecosystems, and we compiled data on the retention and distribution of deposited N from ^15^N tracer experiments. We quantified the retention of ^15^NH_4_^+^ and ^15^NO_3_^−^ among ecosystems and predicted the patterns of C sinks induced by N deposition. The retention of deposited N of NH_x_ and NO_y_ was 25.02 and 14.13 Tg N yr^−1^, respectively. The N‐induced C sink was estimated to be 0.88 Pg C yr^−1^, accounting for approximately 25.48% of the total terrestrial C sink. Although we found that the C sink induced by NH_x_ deposition was greater than that induced by NO_y_ deposition, the contribution of NO_y_ to the terrestrial C sink remains critical because of its greater carbon gain per unit. Therefore, under stable total future N deposition, an increase in the NH_x_/NO_y_ ratio may partially diminish the contribution of N deposition to the terrestrial C sink. These findings underscore the importance of precisely quantifying the fate of NH_x_ and NO_y_ within each pool and changes in the plant C/N ratio for reliably predicting terrestrial C sinks induced by N deposition. However, more relevant evidence of N retention and C emissions in the context of long‐term nitrogen deposition is needed to further evaluate the role of N deposition in the net C sinks of terrestrial ecosystems.

## 5 Materials and Methods

### 5.1 Data Collection and Processing

Peer‐reviewed publications (from 1967 to 2022) for ecosystem‐scale ^15^N tracer studies were searched with the following keywords: (fate^*^ OR retention OR redistribution OR distribution) AND (^15^N tracer* OR ^15^N OR N‐15 OR nitrogen stabilizer OR N stabilizer OR stable isotop^*^) AND (nitrogen OR ammonium OR nitrate) AND (nitrogen recovery OR ^15^N recovery). The criteria for the incorporation of data into our analysis included the following: (1) the study treatment contained at least one ^15^NH_4_^+^, ^15^NO_3_^−^ or ^15^NH_4_^15^NO_3_ experiment, and the recovery was reported as a percentage; (2) the sampling times in each experiment were clearly reported; (3) the ^15^N tracer experiments were carried out in natural terrestrial ecosystems, with studies conducted on crops, monocultures or laboratories excluded; and (4) studies on soil cores that did not include entire plants were excluded. Experiments conducted at multiple sites that were reported in a single article were considered independent studies. On the basis of these criteria, we obtained 829 observations from 87^15^N isotope tracer experiments, of which 60 studies (431 observations) involved labeled ^15^NH_4_^+^, 45 studies (290 observations) involved labeled ^15^NO_3_^−^, and 19 studies (108 observations) involved labeled ^15^NH_4_^15^NO_3_ (Supplementary Dataset). We synthesized observations from studies that included ^15^N recovery, as well as auxiliary data that included information on atmospheric N deposition, ^15^N tracer type, ^15^N tracer addition, sampling time, mean annual temperature (MAT), mean annual precipitation (MAP), biome type and soil physicochemical properties (e.g., soil pH, bulk density, soil silt and clay content, C/N, soil organic carbon (SOC), total nitrogen (TN)), where available (Supplementary Dataset). The original data were obtained directly when presented in tables and Supporting Information or indirectly when shown as figures in the publication using GetData Graph Digitizer 2.26.

The studies included in our analysis reported total ecosystem ^15^N tracer recovery, including ^15^N retained in plant biomass (foliage, branch, stem, bark, fine root, coarse root), litter (Oi layer), soil (organic soil: Oe layer and Oa layer, mineral soil, microbes), leachate, and gases. The total ecosystem recovery time after the application of ^15^N ranged from less than 1 day to 19 years. To analyze ^15^N recovery on a time scale, the duration of the study was divided into four categories on the basis of the time from the application of the tracer to the collection of the data: < 100 days (*n* = 45 studies), 100–500 days (*n* = 62 studies), 500–1000 days (*n* = 24 studies), and > 1000 days (*n* = 14 studies). Studies of multiple applications of ^15^N were classified on the basis of the time of the first application of ^15^N. To evaluate whether tracer duration influenced nitrogen recovery, we examined the relationship between ^15^N recovery and labeling duration across the compiled dataset. As no significant association was identified (Figure S8), ^15^N recovery measurements obtained at different labeling durations were treated as independent observations and directly incorporated into the dataset for subsequent analyses. In addition, the data were pooled into 8 terrestrial biomes for analysis: evergreen broadleaf forest, deciduous broadleaf forest, evergreen coniferous forest, deciduous coniferous forest, mixed forest, grassland, cropland, and others (Figure 1). These biome categories were derived from the ESA Climate Change Initiative (CCI) land cover dataset (https://maps.elie.ucl.ac.be/CCI/viewer/).

### 5.2 Nitrogen Deposition and Retention

Eleven potential factors affecting ecosystem N retention were considered [25, 54, 55, 56], including MAT, MAP, N deposition, annual net primary production (NPP) and various soil properties (pH, bulk density, clay, silt, SOC, TN, and C/N) (Supplementary Dataset), and missing data on potential factors were extracted from global data map products based on the geographical coordinates of the experimental sites (Table S1) as independent variables to estimate the patterns of ^15^N recovery at the global scale. Considering the varied responses of distinct tree tissues to deposited N retention in forest ecosystems, we subdivided tree tissues into woody tissue (stem, branch, coarse root) and nonwoody tissue (foliage, bark, fine root) to increase predictive accuracy with respect to ^15^N recovery. In addition, considering that long‐term N deposition may result in a decrease in the soil C/N ratio [45], we performed sensitivity analysis using the lower limit of the 95% confidence interval for the C/N ratio of organic and mineral soils compared with a standard run with central parameter values. Using three data sources for 2010–2014 N deposition data (derived from the EMEP MSC‐W model, HTAP‐II ensemble, and CMIP6), we estimated the retention of NH_x_ and NO_y_ in different parts of the ecosystem (including plants, organic soils, and mineral soil).

### 5.3 Deposited N‐Induced C Sink Estimation

The stoichiometric upscaling method [10] was used to estimate the N‐induced C sink, which was estimated by multiplying the N retention in each pool by the C/N ratios in those pools. Therefore, the C sink induced by NH_x_ and NO_y_ deposition was calculated as follows [6]:

(1)

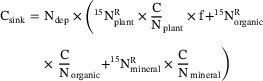

where N_dep_ represents NH_x_ or NO_y_ deposition (g m^−2^ year^−1^); 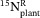, 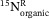 and 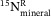 represent the recoveries of NH_x_ and NO_y_ in plant, organic soil, and mineral soil, respectively; ${\frac{C}{N}}_{\mathrm{plant}}$, ${\frac{C}{N}}_{\mathrm{organic}}$ and ${\frac{C}{N}}_{\mathrm{mineral}}$ represent the C/N ratios of plant biomass, organic soil, and mineral soil, respectively; and f represents the flexible C/N ratio that explains plant N absorption in response to increased N deposition. Owing to a lack of reports on plant and soil C/N ratios, we calculated the C/N ratios of ecosystem plants and organic soil by collecting the mean values in each ecosystem from different studies (Table S7), and missing mineral soil C/N ratios were obtained from the World Soil Database (Table S1). The C gain for a given unit of N deposition is defined as the additional mass of C sequestered per unit mass of N deposited and is an indicator of the efficiency with which ecosystems take up N [18]. The C gain from N deposition can be estimated by dividing N‐induced C sequestration by the total N deposition in each ecosystem.

### 5.4 Ecosystem N Status Thresholds

Considering that the ecological effects induced by different N deposition rates vary and that the response of plant N absorption to increased N deposition in different ecosystems may have thresholds [14, 57], we used flexible stoichiometry to assess the effect of N deposition on plant N absorption [58]. Research has demonstrated that N deposition can alleviate N limitation in ecosystems and stimulate plant N absorption [59]. However, an increase in N deposition weakens plant N limitation, which diminishes the C response to N input [14]. When the N deposition rate is excessively high, this stimulating effect decreases or even vanishes due to soil acidification and nutrient imbalances [60]. Therefore, on the basis of the ratio of nitrogen resorption efficiency (NRE) and phosphorus resorption efficiency (PRE) in the leaves of dominant species on a global scale [15], we used the f value to explain the response of plants to increased N deposition. Simply put, when NRE_dom_/PRE_dom_ > 0.16, indicating N limitation, we assume that N deposition has no effect on the C/N ratios of plants (f = 1); when NRE_dom_/PRE_dom_ < 0.16, we set f to 0.5; and when NRE_dom_/PRE_dom_ < −0.16, we assume that N deposition no longer promotes plant N absorption (f = 0). In addition, 80% of the N retained by the soil organic layer is assumed to be fixed in the persistent soil organic matter (SOM) pool to increase the soil C sink [6].

### 5.5 Predictive Modeling and Uncertainty

The distribution patterns of global ^15^N recovery and N‐induced C sink were also predicted by using four linear regression models (the multiple linear regression model, multiple stepwise regression model, least angle regression model, and elastic net model) and four nonlinear regression models (the cubist model, boosted tree model, bagged tree model, and random forest model). All models except the linear regression model and bagged tree model have a built‐in feature selection process and can be individually adjusted to improve model accuracy and control model complexity [61]. To mitigate the impact of multicollinearity, we estimated the variance inflation factor (VIF) for all predicted independent variables. We eliminated variables with the highest VIF until the VIF for all independent variables was below five [62]. All models were evaluated using a fivefold cross‐validation method [63], in which the entire dataset was randomly divided into five groups, 80% of the data were trained, and 20% of the data were validated to assess uncertainty in the model structure. The root mean square error (RMSE) and *R* ^2^ of all models were estimated as criteria for ranking model performance [64]. We use a 95% confidence interval to represent uncertainty in the prediction model.

## Author Contributions

L.L. designed the research. M.X. and N.J. performed the research. M.X., N.J., and L.M. analyzed the data. M.X. and L.L. wrote the paper. M.X., L.L., L.M., Q.Y., F.Z., and X.L. revised the paper.

## Funding

This work was supported by the National Natural Science Foundation of China (42471077), Natural Science Foundation of Xinjiang Uygur Autonomous Region (2022D01E100), and Youth Innovation Promotion Association of the Chinese Academy of Sciences (2020434).

## Conflicts of Interest

The authors declare no conflicts of interest.

## Supporting information




**Supporting File 1**: advs75019‐sup‐0001‐SuppMat.docx

**Supporting File 2**: advs75019‐sup‐0002‐Data.xlsx

**Supporting File 3**: advs75019‐sup‐0003‐NHXCsink.tif

**Supporting File 4**: advs75019‐sup‐0004‐NOYCsink.tif

## Data Availability

The data that support the findings of this study are available in the supplementary material of this article.
