# Supplementary material for: Global Nitrogen Deposition Promotes Carbon Sink Formation in Terrestrial Ecosystems
Source: Adv Sci (Weinh). 2026 Mar 25;13(33):e20069. doi: 10.1002/advs.202520069 (PMC13271591; doi:10.1002/advs.202520069)
Supplement: Supplementary file 1 — Supporting File 1: advs75019‐sup‐0001‐SuppMat.docx [file ADVS-13-e20069-s003.docx]

Supporting Information

Global deposition of nitrogen induces carbon sinks in terrestrial ecosystems

*Lei Li^*^,* *Ming-Yu Xie, Nan Jia,* *Li-Dong Mo,* *Qiang Yu, Fan-Jiang Zeng, Xiang-Yi Li*

Lei Li and Ming-Yu Xie contributed equally to this work.

**This PDF file includes:**

Supporting text

Figures S1 to S13

Tables S1 to S12


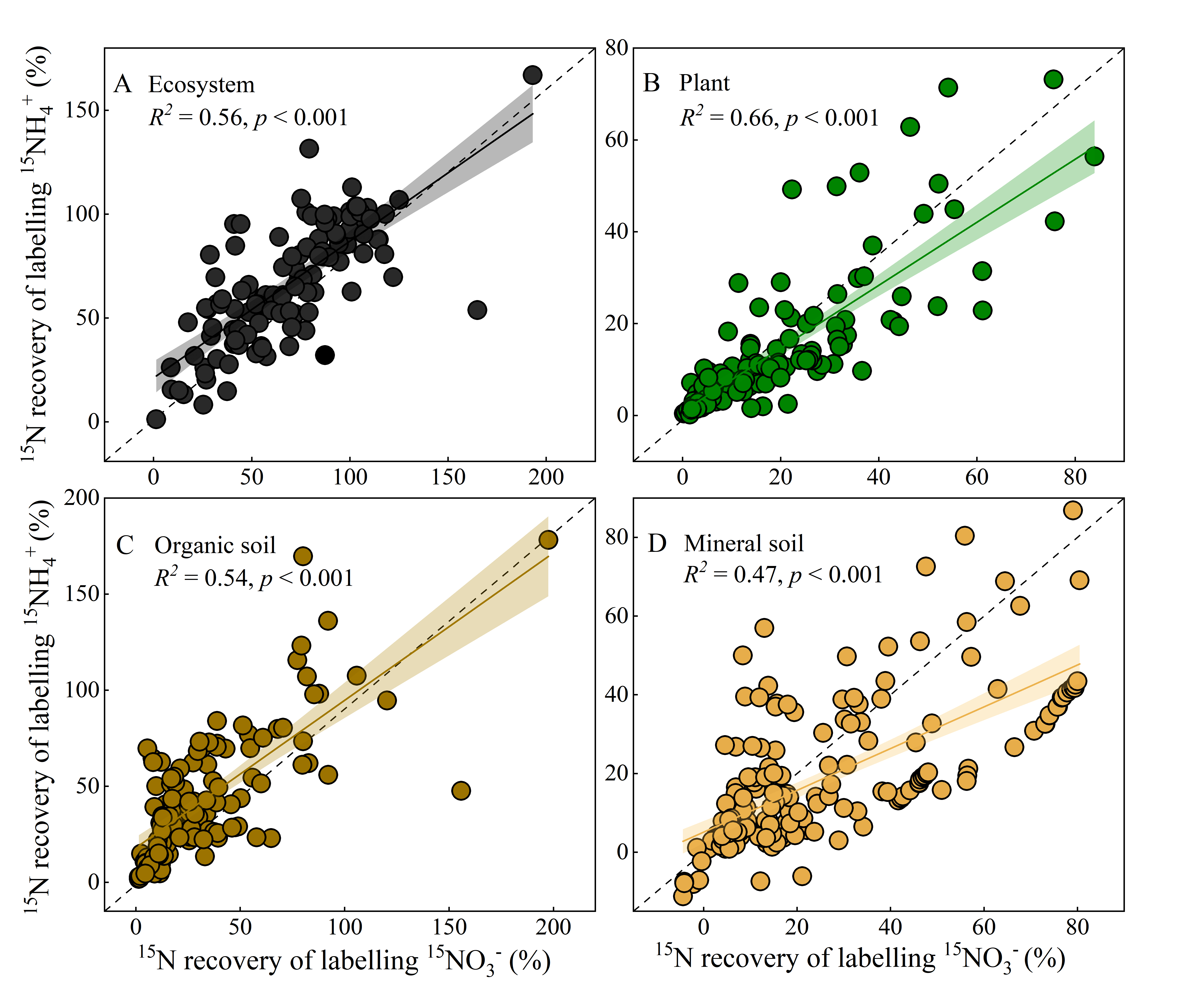


**Fig. S1.** Relationship between mean ^15^N recovery of ^15^NH_4_^+^ tracer and ^15^NO_3_^-^ tracer in the whole ecosystem (A), plants (B), organic soil (C) and mineral soil (D). The black dotted line indicates that the ^15^N recovery of ^15^NH_4_^+^ tracer and ^15^NO_3_^-^ tracer is 1:1. The shaded region represents 95% confidence interval of linear regression for each pool. The slope of the relationship for the whole ecosystem, plant, organic soil, and mineral soil was 0.66, 0.69, 0.78, 0.53, respectively.


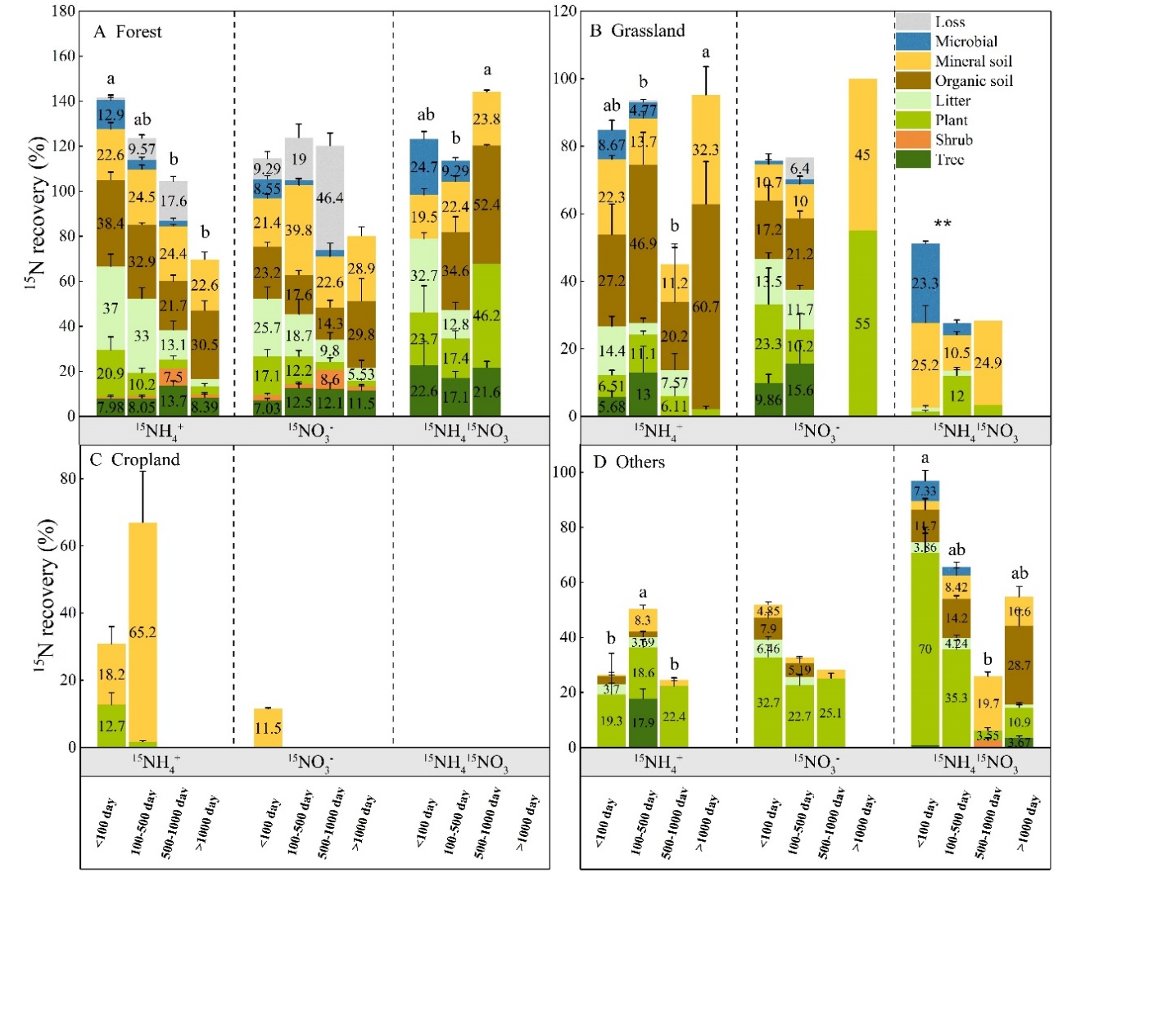


**Fig. S2.** Total ^15^N recovery of individual pools from forest (A), grassland (B), cropland (C) and other ecosystem (D) in different periods. Signiﬁcant differences among different forest types are indicated by different lettering (p < 0.05). **p < 0.01.

**
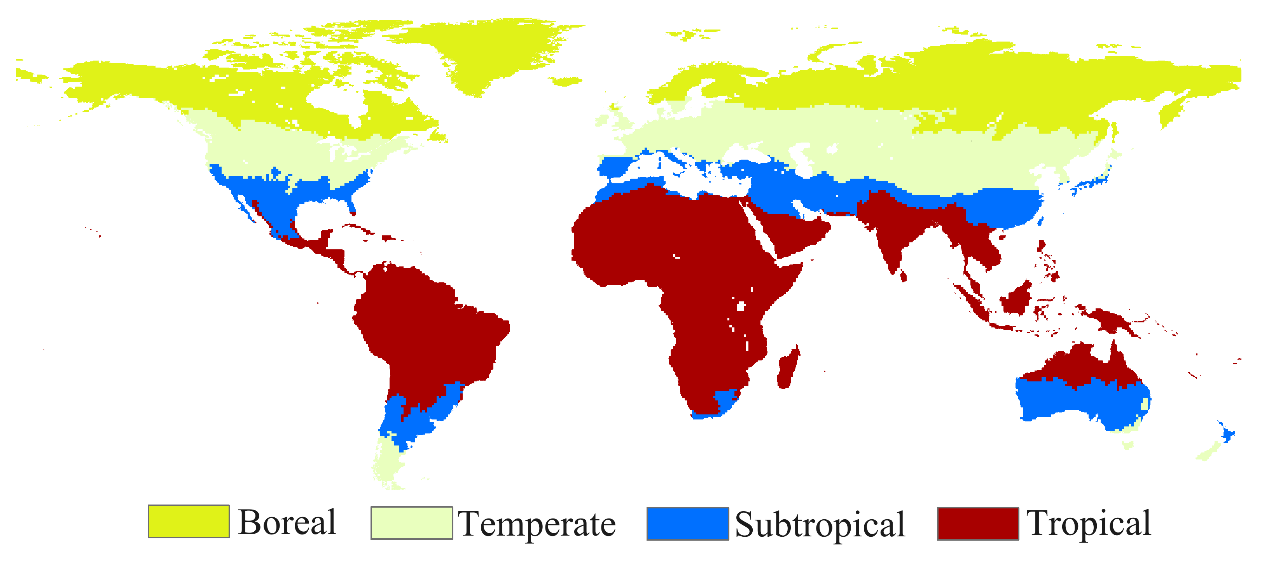
**

**Fig. S3**. Global Ecological Zones.


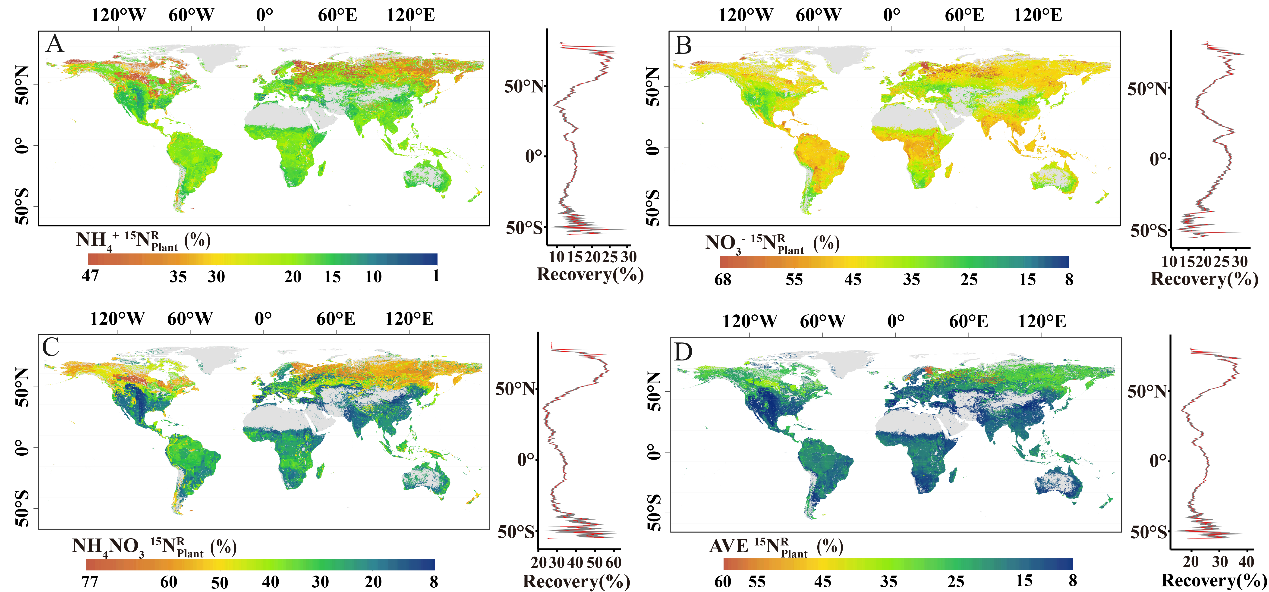


**Fig. S4**. Modelled global patterns of plant N retention of ^15^NH_4_^+^ (A), ^15^NO_3_^-^ (B), ^15^NH_4_^15^NO_3_ (C) and their average value (D) in terrestrial ecosystems. Values were predicted using a data-driven random forest model with global climate, vegetation and soil properties. The red shadows represent the 5th and 95th quantile, representing the 95% prediction interval of the random forest model.


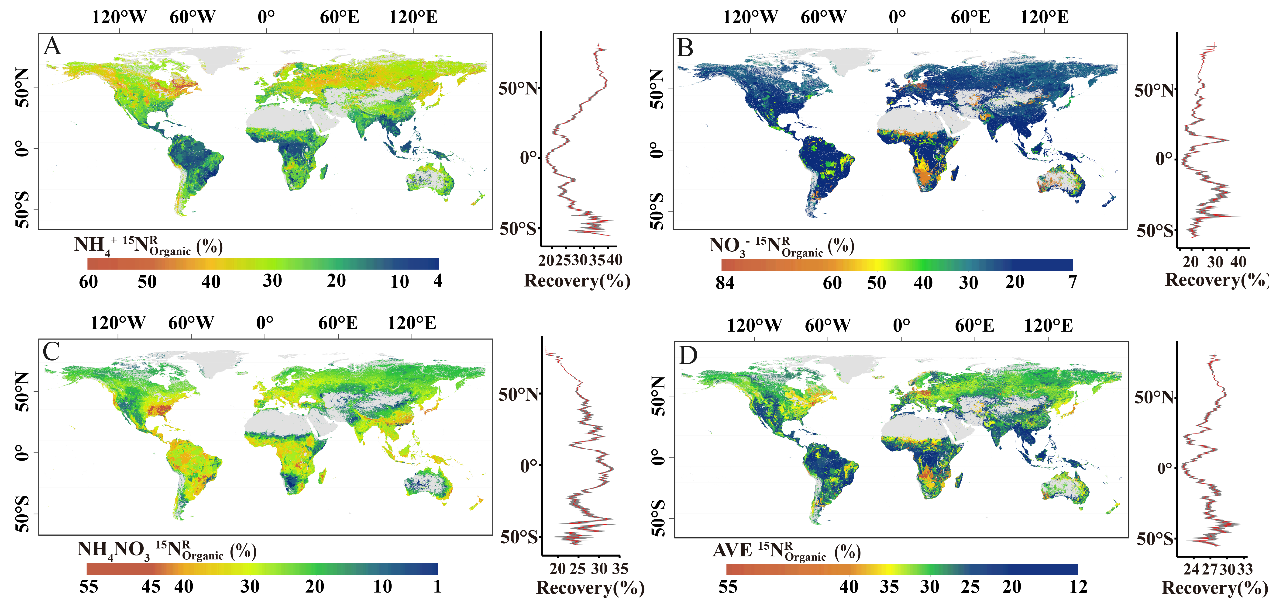


**Fig. S5**. Modelled global patterns of organic soil N retention of ^15^NH_4_^+^ (A), ^15^NO_3_^-^ (B), ^15^NH_4_^15^NO_3_ (C) and their average value (D) in terrestrial ecosystems. Values were predicted using a data-driven random forest model with global climate, vegetation and soil properties. The red shadows represent the 5th and 95th quantile, representing the 95% prediction interval of the random forest model.


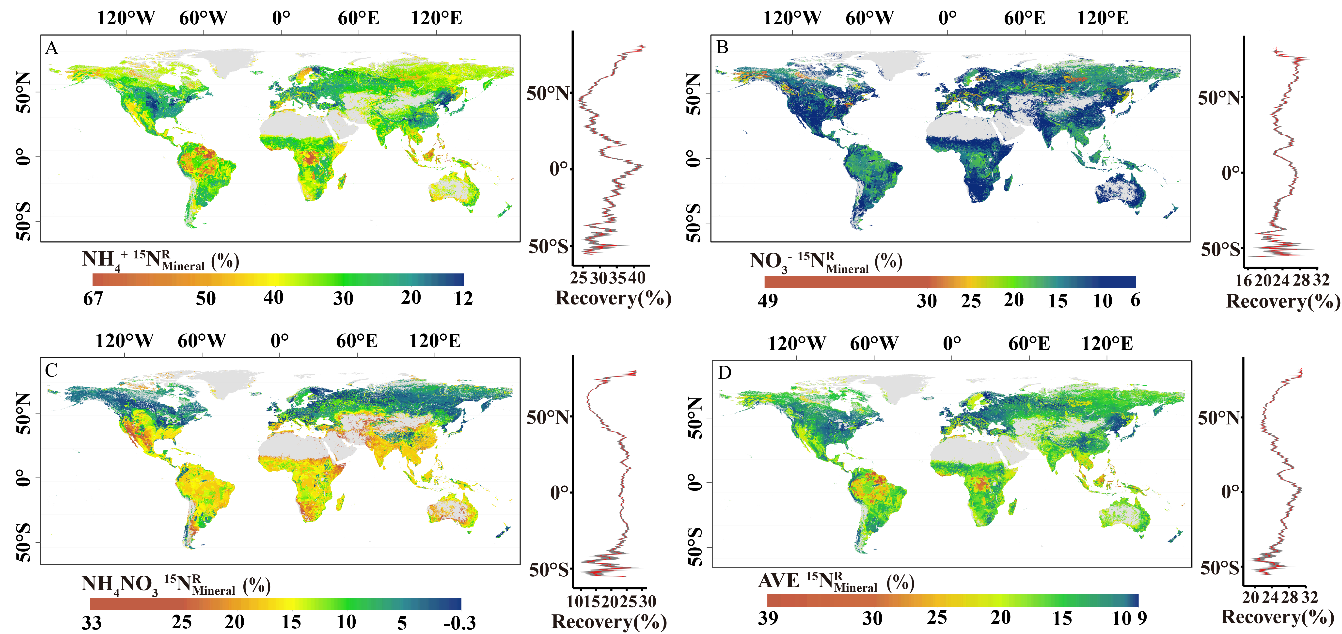


**Fig. S6**. Modelled global patterns of mineral soil N retention of ^15^NH_4_^+^ (A), ^15^NO_3_^-^ (B), ^15^NH_4_^15^NO_3_ (C) and their average value (D) in terrestrial ecosystems. Values were predicted using a data-driven random forest model with global climate, vegetation and soil properties. The red shadows represent the 5th and 95th quantile, representing the 95% prediction interval of the random forest model.


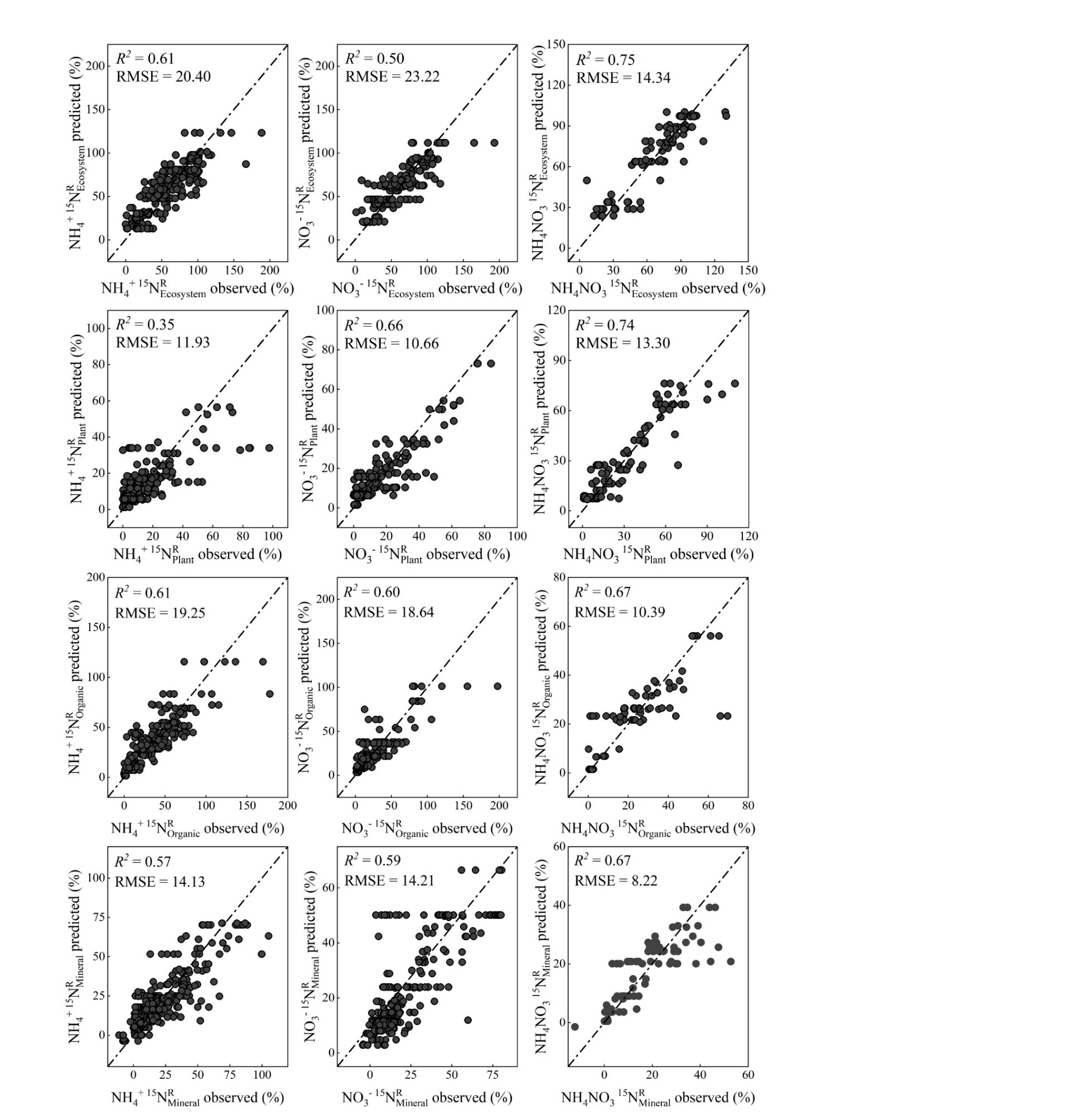


**Fig. S7**. The ecosystem recovery model verification. Relationship between observed and predicted values in ecosystem, plant, organic soil and mineral soil. The dotted line is the 1:1 line between observed and predicted value.

**
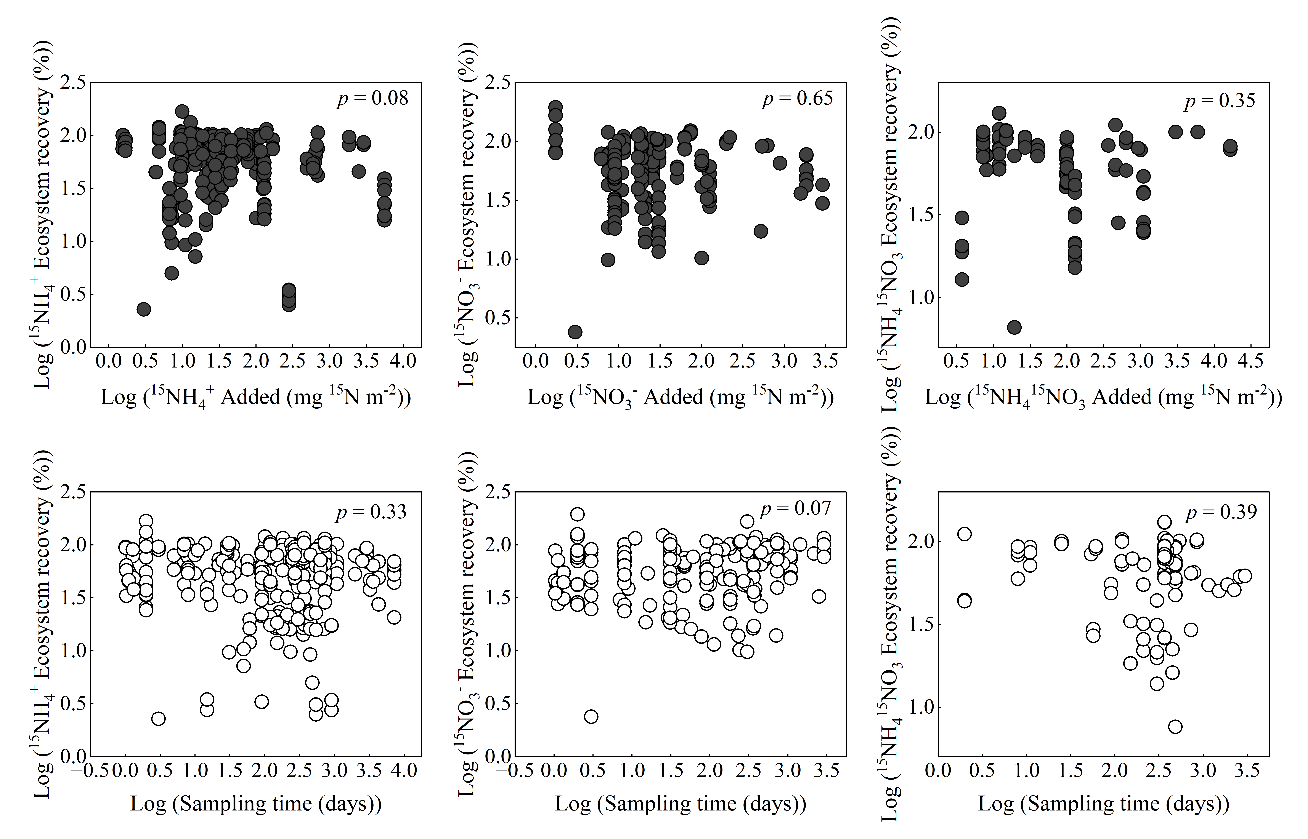
**

**Fig. S8**. Correlation analysis between the ^15^N recovery and the amount of ^15^N applied and sampling time. The data were showed as logarithmic transformations.


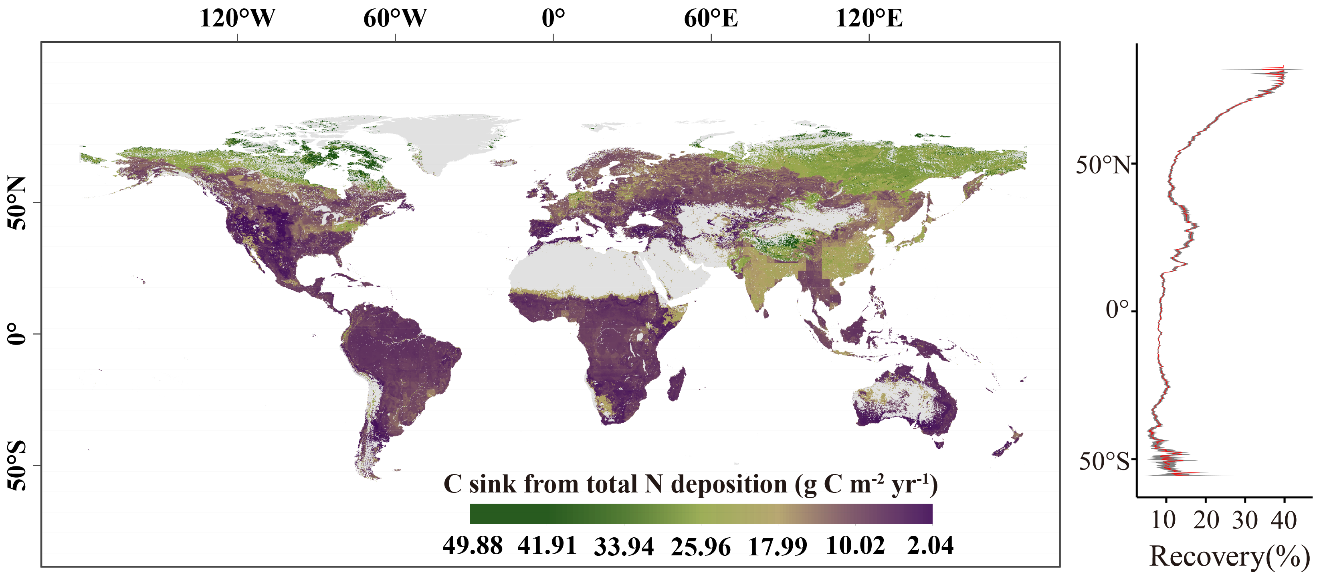


**Fig. S9.** Spatial pattern and relative uncertainty of the terrestrial C sink induced by N deposition. The values were predicted using a data-driven random forest model with global climate, vegetation and soil properties. The grey shadows represent the 5th and 95th quantiles, which represent the 95% prediction interval of the random forest model.


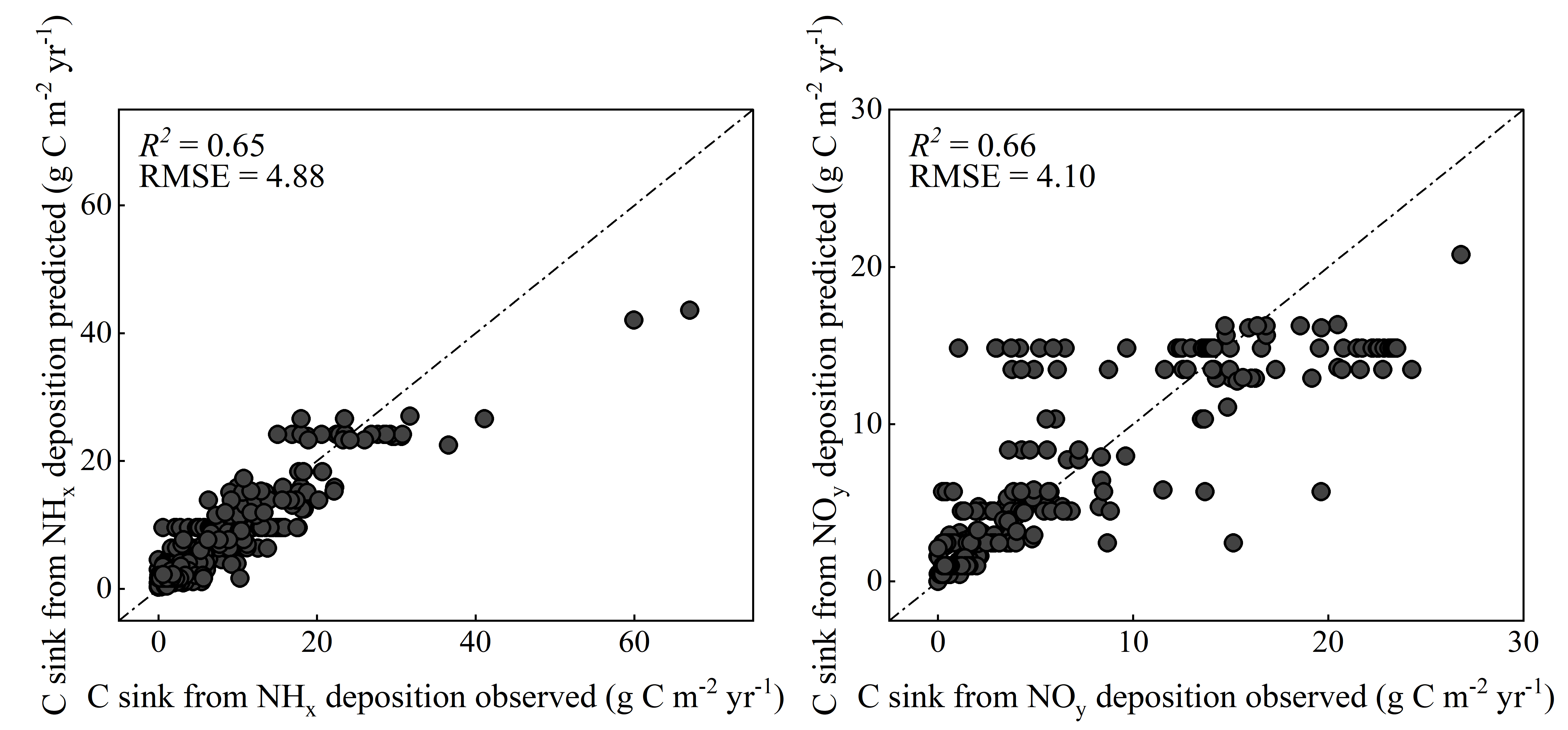


**Fig. S10**. The N-induced C sink model verification. Relationship between observed and predicted values in NH_x_ and NO_y_ induced C sink. The dotted line is the 1:1 line between observed and predicted value.

**
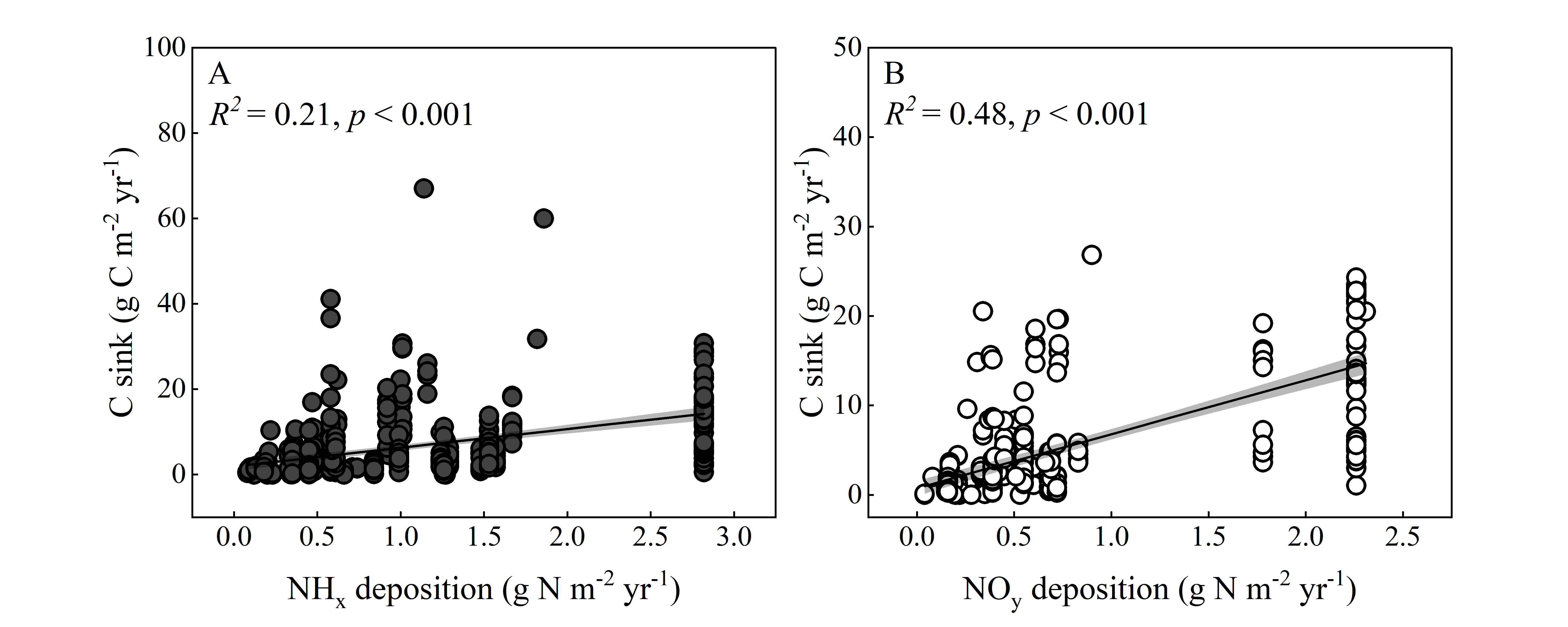
**

**Fig. S11**. The relationship between measured data of NH_x_ deposition (A) and NO_y_ deposition (B) with their induced C sink. The solid line is the linear regression line and the shaded region represents the 95% confidence interval for the relationship.


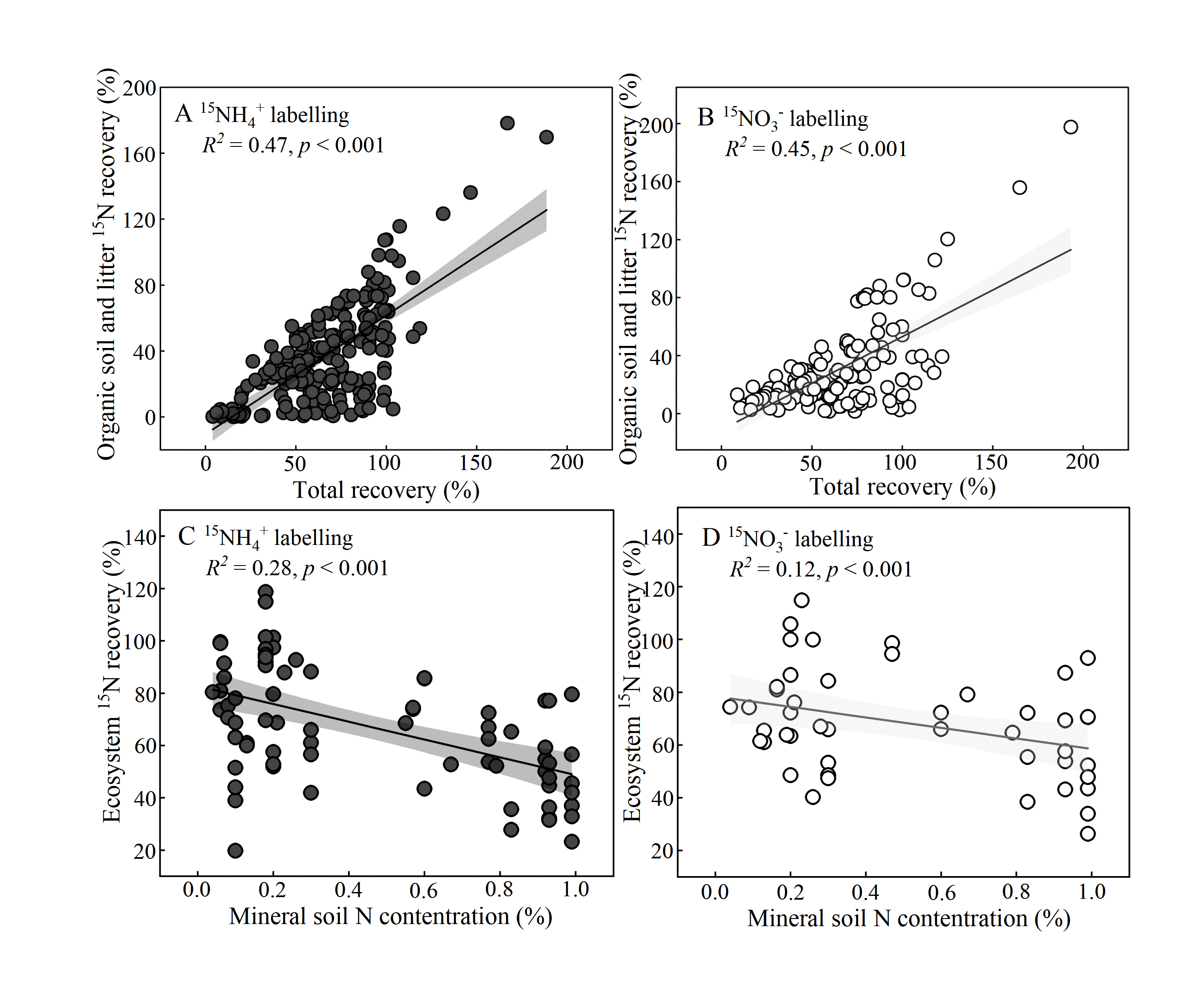


**Fig. S12**. Linear regression analysis of the soil organic layer (A, B) and the C/N ratio of mineral soil (C, D) with total ecosystem ^15^N recovery when labeling ^15^NH_4_^+^ and ^15^NO_3_^-^. The solid line is the linear regression line and the shaded region represents the 95% confidence interval for the relationship.


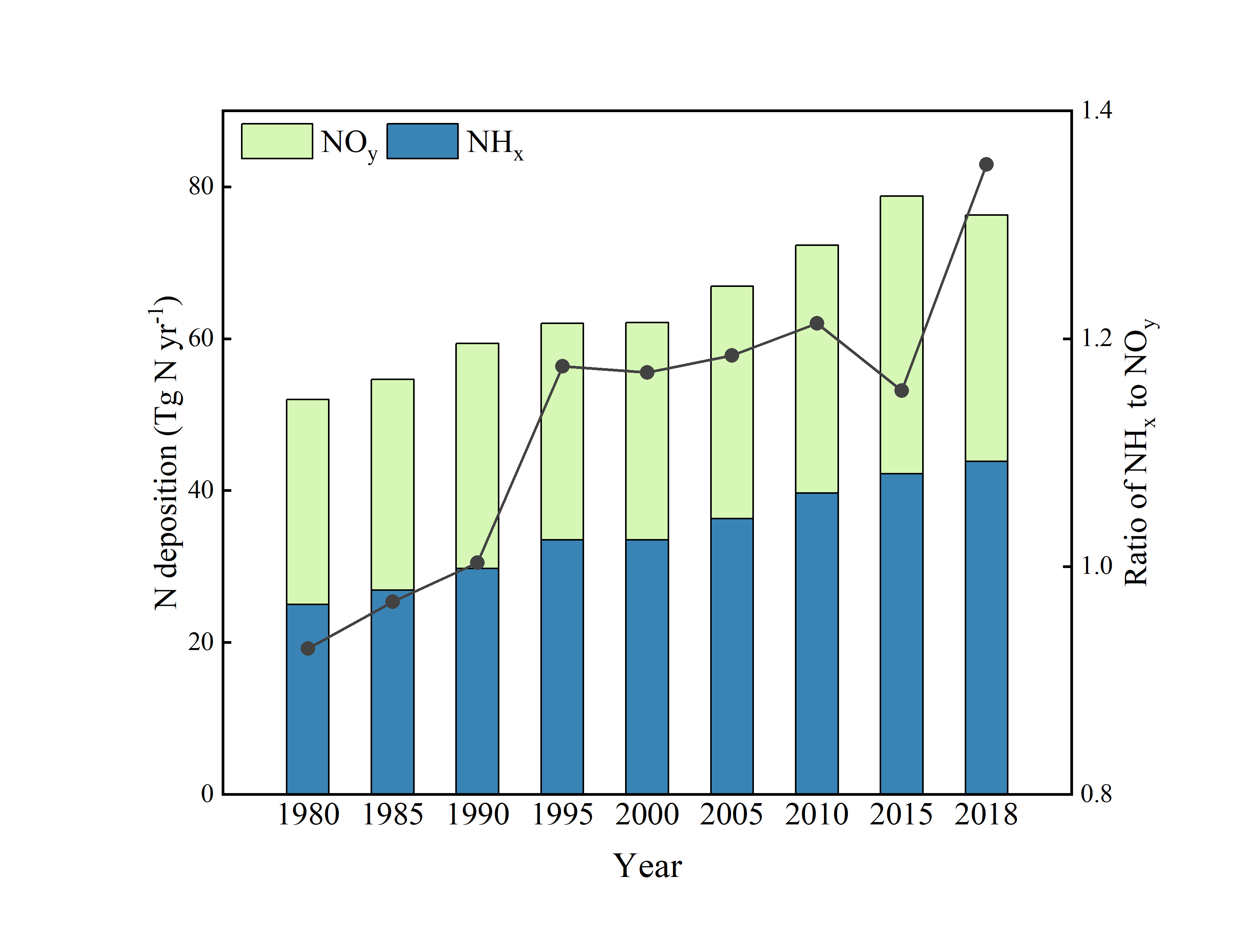


**Fig. S13**. Temporal variation of NH_x_ and NO_y_ depositions and their ratio from 1980 to 2018.

**Table S1**. Gridded data used in this study.

| Type | Variable | Unit | Original resolution | Data source |
| --- | --- | --- | --- | --- |
| Climate | Mean annual temperature | ℃ | 1 km^2^ | http://worldclim.org/bioclim |
|  | Mean annual precipitation  N deposition (2010) | mm  mg m^-2^ yr^-1^ | 1 km^2^  1^°^ | <http://worldclim.org/bioclim>  https://thredds.met.no/thredds/catalog/data/EMEP/Articles_data/Schwede_etal_Ndep_2018/catalog.html |
|  | N deposition (2010) | mg m^-2^ yr^-1^ | 1^°^ | https://thredds.met.no/thredds/catalog/data/EMEP/Articles_data/Schwede_etal_Ndep_2018/catalog.html |
|  | N deposition (2010-2014) | kg m^-2^ s^-1^ | 250 km | https://esgf-data.dkrz.de/search/cmip6-dkrz/ |
| Vegetation | Net Primary Production  (1981-2018) | g m^-2^ d^-1^ | 5000m | http://www.geodata.cn |
| Soil properties | Soil organic carbon content | g kg^-1^ | 1000 m | https://data.isric.org/geonetwork/srv/eng/catalog.search#/metadata/dc7b283a-8f19-45e1-aaed-e9bd515119bc |
|  | Soil total nitrogen | g kg^-1^ | 1000 m | https://data.isric.org/geonetwork/srv/eng/catalog.search#/metadata/dc7b283a-8f19-45e1-aaed-e9bd515119bc |
|  | Soil pH |  | 1000 m | https://data.isric.org/geonetwork/srv/eng/catalog.search#/metadata/dc7b283a-8f19-45e1-aaed-e9bd515119bc |
|  | Soil silt | % | 1000 m | https://data.isric.org/geonetwork/srv/eng/catalog.search#/metadata/dc7b283a-8f19-45e1-aaed-e9bd515119bc |
|  | Soil clay | % | 1000 m | https://data.isric.org/geonetwork/srv/eng/catalog.search#/metadata/dc7b283a-8f19-45e1-aaed-e9bd515119bc |
|  | Soil bulk density | kg dm^-3^ | 1000 m | https://data.isric.org/geonetwork/srv/eng/catalog.search#/metadata/dc7b283a-8f19-45e1-aaed-e9bd515119bc |

**Table S2** The ^15^N recovery of ^15^N, ^15^NH_4_^+^, ^15^NO_3_^-^ and ^15^NH_4_^15^NO_3_ in different pools for different types of forest. The ^15^N recovery are reported as mean±se. Signiﬁcant differences among different forest types are indicated by different lettering (*p* < 0.05).

| **Ecosystem type** | **^15^N recovery (%)** | | | | | | | | |
| --- | --- | --- | --- | --- | --- | --- | --- | --- | --- |
|  | **Tree** | **Shrub** | **Grass** | **Litter** | **Organic soil** | **Mineral soil** | **Microbial biomass** | **Loss** | **Total (excluding loss)** |
| Deciduous broadleaf forest | 5.04±1.19 | \ | 2.58±1.19c | 14.17±2.52ab | 16.99±3.01bc | 13.31±2.18ab | 3.50±1.33ab | \ | 47.45±4.42b |
| Evergreen broadleaf forest | 14.18±3.25 | 0.08±0.02b | 7.45±1.23bc | 24.91±9.00ab | 12.86±2.49c | 23.93±4.23a | 0.28±0.02b | \ | 59.86±3.82b |
| Deciduous coniferous forest | 6.37±0.99 | 2.06±0.31a | 9.15±3.42ab | \ | 26.82±4.82a | 10.21±5.23b | \ | 0.11±0.06b | 77.34±7.07a |
| Evergreen coniferous forest | 17.39±2.65 | 1.72±1.22a | 21.51±4.84a | 25.67±4.60a | 23.61±3.19bc | 22.19±3.29a | 5.13±1.41ab | 4.61±2.16ab | 57.48±2.62b |
| Mixed forest | 12.60±2.08 | 1.72±0.29a | 4.85±1.28bc | 19.46±2.79b | 41.72±3.03ab | 25.26±2.14a | 12.02±2.36a | 8.59±2.33a | 51.88±1.76b |
| **^15^NH_4_^+^** | | | | | | | | | |
| Deciduous broadleaf forest | 6.38±1.88 | \ | 1.97±0.87b | 30.98±4.30 | 30.83±3.60bc | 22.40±3.92 | 6.14±2.40ab | \ | 60.57±5.38ab |
| Evergreen broadleaf forest | 12.22±1.87 | 0.05±0.01b | 7.21±1.64b | 38.17±12.84 | 27.93±5.42c | 21.48±6.04 | 0.83±0.05b | \ | 70.06±5.64ab |
| Deciduous coniferous forest | 4.67±1.32 | 1.76±0.49ab | 10.00±3.93ab | \ | 49.35±8.88a | 12.88±6.45 | \ | 0.16±0.06 | 76.80±10.21a |
| Evergreen coniferous forest | 9.95±1.05 | 2.49±1.67a | 22.60±5.13a | 33.26±4.99 | 27.30±1.98c | 25.43±2.43 | 7.49±1.70ab | 9.87±3.00 | 60.88±3.40ab |
| Mixed forest | 10.73±1.94 | 1.54±0.41ab | 6.34±1.36b | 25.58±4.26 | 46.70±4.63ab | 23.15±1.77 | 11.74±2.57a | 14.42±3.96 | 47.67±2.82b |
| **^15^NO_3_^-^** | | | | | | | | | |
| Deciduous broadleaf forest | 8.75±1.70b | \ | 5.78±2.69b | 11.53±3.27b | 20.14±5.42ab | 17.52±2.62b | 4.35±1.58 | \ | 30.92±6.07c |
| Evergreen broadleaf forest | 20.45±5.68a | 0.18±0.06b | 5.25±0.90b | 30.45±13.00ab | 10.65±2.04b | 22.32±4.11b | \ | \ | 60.32±7.03ab |
| Deciduous coniferous forest | 14.44±1.65ab | 4.42±0.46a | 17.44±6.34ab | \ | 31.12±5.59a | 17.75±9.25b | \ | 0.18±0.13 | 77.98±10.89a |
| Evergreen coniferous forest | 11.90±1.71ab | 2.67±1.98a | 19.93±3.41a | 43.74±8.81a | 22.59±2.17ab | 24.35±3.30b | 7.89±2.54 | 17.80±6.47 | 50.21±4.44bc |
| Mixed forest | 6.90±1.01b | 3.62±0.45a | 8.22±2.49ab | 6.69±1.10b | 21.95±2.22ab | 34.06±2.37a | 8.90±1.82 | 37.13±9.50 | 52.00±2.17bc |
| **^15^NH_4_^15^NO_3_** | | | | | | | | | |
| Deciduous broadleaf forest | \ | \ | \ | \ | \ | \ | \ | \ | \ |
| Evergreen broadleaf forest | 9.85±2.19b | \ | 9.88±1.13 | 6.10±1.16 | \ | 27.98±2.54a | \ | \ | 43.16±5.20b |
| Deciduous coniferous forest | \ | \ | \ | \ | \ | \ | \ | \ | \ |
| Evergreen coniferous forest | 30.32±5.18a | \ | 21.99±6.00 | \ | 20.93±5.43 | 16.79±4.14b | \ | \ | 76.51±4.75a |
| Mixed forest | 20.17±3.28ab | \ | \ | 26.13±3.00 | 56.50±2.24 | 18.56±2.29b | 15.44±2.69 | \ | 88.44±3.36a |

**Table S3** The ^15^N recovery of ^15^N, ^15^NH_4_^+^, ^15^NO_3_^-^ and ^15^NH_4_^15^NO_3_ in woody and non-woody tissues of forest trees. The ^15^N recovery are reported as mean±se. Signiﬁcant differences among different forest types are indicated by different lettering (*p* < 0.05).

| **Ecosystem type** | **^15^N recovery (%)** | | | | | | |
| --- | --- | --- | --- | --- | --- | --- | --- |
|  | **Tree foliage** | **Tree bark** | **Tree fine root** | **Tree stem** | **Tree coarse root** | **Tree branch** | **Total** |
| Deciduous broadleaf forest | 2.222±0.004b | 1.055±0.002 | 5.071±0.010ab | 2.441±0.007ab | 3.597±0.005 | 4.060 | 7.393±0.012b |
| Evergreen broadleaf forest | 3.708±0.005b | 1.253±0.002 | 5.034±0.014ab | 1.103±0.002b | 1.740±0.002 | 4.116±0.006 | 10.013±0.018a |
| Deciduous coniferous forest | 2.523±0.006b | 1.620±0.004 | 2.344±0.007b | 0.972±0.005b | 1.860±0.004 | 1.500±0.003 | 13.711±0.019ab |
| Evergreen coniferous forest | 7.115±0.009ab | 6.044±0.034 | 5.854±0.007a | 3.459±0.003a | 2.750±0.019 | 4.615±0.012 | 12.355±0.012ab |
| Mixed forest | 8.699±0.023a | 2.594±0.010 | 6.005±0.006a | 2.466±0.005ab | 2.589±0.005 | 3.663±0.011 | 9.596±0.010ab |
| **^15^NH_4_^+^** | | | | | | | |
| Deciduous broadleaf forest | 1.341±0.002 | 0.825±0.003 | 5.482±0.017ab | 3.175±0.013a | \ | \ | 6.369±1.784 |
| Evergreen broadleaf forest | 3.085±0.005 | 1.213±0.001 | 4.985±0.015ab | 1.139±0.001ab | 1.660±0.002 | 2.587±0.005 | 4.700±0.704 |
| Deciduous coniferous forest | 1.000±0.003 | 0.700±0.002 | 1.160±0.002b | 0.300±0.002b | 0.840±0.02 | 0.820±0.002 | 12.653±2.261 |
| Evergreen coniferous forest | 5.618±0.008 | 0.865±0.003 | 4.659±0.007ab | 2.622±0.003ab | 0.733±0.003 | 3.822±0.017 | 9.909±1.897 |
| Mixed forest | 7.560±0.055 | 0.920±0.002 | 7.694±0.011a | 2.489±0.007ab | 2.900±0.008 | 2.480±0.004 | 10.378±1.177 |
| **^15^NO_3_^-^** | | | | | | | |
| Deciduous broadleaf forest | 3.513±0.010b | 1.253±0.004b | 4.591±0.008 | 1.707±0.005ab | 3.597±0.005 | 4.060 | 8.732±1.323b |
| Evergreen broadleaf forest | 3.487±0.008b | 1.472±0.004b | 7.764±0.049 | 1.570±0.003ab | 1.820±0.002 | 5.721±0.012 | 14.440±1.654a |
| Deciduous coniferous forest | 3.792±0.008b | 2.540±0.005b | 3.331±0.011 | 1.475±0.009ab | 2.880±0.005 | 2.180±0.004 | 20.502±5.486ab |
| Evergreen coniferous forest | 6.993±0.010ab | 19.474±0.105a | 4.835±0.005 | 4.043±0.007a | 3.960±0.030 | 7.125±0.024 | 11.909±2.030ab |
| Mixed forest | 10.124±0.026a | 1.909±0.004b | 4.146±0.006 | 1.040±0.005b | 2.144±0.005 | 0.750 | 6.905±1.021b |
| **^15^NH_4_^15^NO_3_** | | | | | | | |
| Deciduous broadleaf forest | \ | \ | \ | \ | \ | \ | \ |
| Evergreen broadleaf forest | 4.601±0.011 | 0.843±0.001 | 3.044±0.008c | 0.304±0.001b | \ | 5.475±0.024 | 9.853±2.237b |
| Deciduous coniferous forest | \ | \ | \ | \ | \ | \ | \ |
| Evergreen coniferous forest | 16.729±0.082 | 0.950±0.005 | 22.480±0.021a | 7.014±0.014a | \ | 5.675±0.007 | 30.320±5.182a |
| Mixed forest | 7.084±0.020 | 4.217±0.021 | 9.234±0.025b | 3.600±0.013ab | \ | 5.134±0.020 | 20.161±4.116ab |

**Table S4** Comparison of the performance of different models for predicting ecosystem N retention. We applied four linear regression models and four nonlinear models. *R^2^* and root mean square error (RMSE) were used to evaluate the performance of the models.

| Labelled | Model | Ecosystem | | Plant | | Organic soil | | Mineral soil | |
| --- | --- | --- | --- | --- | --- | --- | --- | --- | --- |
|  |  | *R^2^* | RMSE | *R^2^* | RMSE | *R^2^* | RMSE | *R^2^* | RMSE |
| ^15^NH_4_^+^ | Linear regression | 0.10 | 30.78 | 0.13 | 13.53 | 0.22 | 26.90 | 0.17 | 19.71 |
|  | Multiple stepwise regression | 0.12 | 30.41 | 0.15 | 13.22 | 0.12 | 28.10 | 0.14 | 19.96 |
|  | Least angle regression | 0.09 | 31.01 | 0.13 | 13.74 | 0.16 | 27.70 | 0.14 | 19.99 |
|  | Elastic net model  Cubist | 0.09 | 30.97 | 0.13 | 13.57 | 0.16 | 27.71 | 0.15 | 19.90 |
|  |  | 0.59 | 20.85 | 0.33 | 12.42 | 0.55 | 20.62 | 0.55 | 15.03 |
|  | Boosted tree | 0.58 | 20.96 | 0.32 | 12.04 | 0.59 | 19.84 | 0.54 | 14.62 |
|  | Bagged tree  Random forest | 0.51 | 22.55 | 0.30 | 12.30 | 0.56 | 20.07 | 0.47 | 15.76 |
|  |  | 0.61 | 20.40 | 0.35 | 11.93 | 0.61 | 19.25 | 0.57 | 14.13 |
| ^15^NO_3_^-^ | Linear regression | 0.34 | 26.41 | 0.50 | 12.95 | 0.35 | 23.76 | 0.22 | 19.62 |
|  | Multiple stepwise regression | 0.20 | 28.75 | 0.27 | 15.53 | 0.25 | 25.53 | 0.12 | 20.79 |
|  | Least angle regression | 0.31 | 27.21 | 0.47 | 13.38 | 0.29 | 24.72 | 0.19 | 19.96 |
|  | Elastic net model | 0.31 | 27.13 | 0.47 | 13.38 | 0.30 | 24.67 | 0.20 | 19.92 |
|  | Cubist | 0.50 | 23.88 | 0.65 | 10.65 | 0.58 | 19.36 | 0.54 | 15.63 |
|  | Boosted tree | 0.41 | 24.98 | 0.64 | 10.81 | 0.51 | 20.40 | 0.57 | 14.68 |
|  | Bagged tree | 0.42 | 24.72 | 0.49 | 13.37 | 0.53 | 20.10 | 0.51 | 15.60 |
|  | Random forest | 0.50 | 23.32 | 0.66 | 10.66 | 0.60 | 18.64 | 0.59 | 14.21 |
| ^15^NH_4_^15^NO_3_ | Linear regression | 0.35 | 24.18 | 0.58 | 17.19 | 0.49 | 15.97 | 0.44 | 10.77 |
|  | Multiple stepwise regression | 0.39 | 22.39 | 0.62 | 15.92 | 0.49 | 13.50 | 0.44 | 10.72 |
|  | Least angle regression | 0.31 | 23.89 | 0.59 | 16.94 | 0.49 | 13.61 | 0.45 | 10.90 |
|  | Elastic net model | 0.32 | 23.75 | 0.60 | 16.54 | 0.52 | 12.72 | 0.45 | 10.83 |
|  | Cubist | 0.73 | 14.80 | 0.74 | 14.09 | 0.65 | 11.03 | 0.65 | 8.81 |
|  | Boosted tree | 0.75 | 14.60 | 0.72 | 13.94 | 0.65 | 10.93 | 0.66 | 8.61 |
|  | Bagged tree | 0.69 | 16.21 | 0.71 | 14.09 | 0.59 | 11.58 | 0.62 | 8.93 |
|  | Random forest | 0.75 | 14.34 | 0.74 | 13.30 | 0.67 | 10.39 | 0.67 | 8.22 |

**Table S5** Mean deposition and retention of NH_x_ and NO_y_ in the global ecosystem of plants, organic soil and mineral soil pools. N retention was calculated from the deposition and retained fractions of NH_x_ and NO_y_ in each biome. The numbers in brackets represent 95% confidence intervals.

| Ecosystem type | N deposition (Tg N yr^-1^) | N retention (Tg N yr^-1^) | | | |
| --- | --- | --- | --- | --- | --- |
|  |  | Plant | Organic soil | Mineral soil | Ecosystem |
| **NH_x_** |  |  |  |  |  |
| Deciduous broadleaf forest | 3.20 | 0.49(0.20, 2.97) | 0.93(0.41, 6.06) | 0.83(0.39, 4.74) | 2.25(1.00, 13.77) |
| Evergreen broadleaf forest | 3.62 | 0.57(0.18, 2.84) | 0.66(0.21, 3.57) | 1.35(0.46, 5.42) | 2.58(0.85, 11.83) |
| Deciduous coniferous forest | 1.14 | 0.27(0.08, 0.81) | 0.43(0.16, 1.43) | 0.39(0.16, 1.12) | 1.09(0.40, 3.36) |
| Evergreen coniferous forest | 2.02 | 0.38(0.11, 2.76) | 0.69(0.26, 5.34) | 0.53(0.22, 4.30) | 1.60(0.59, 12.40) |
| Mixed forest | 0.29 | 0.05(0.05, 0.46) | 0.10(0.09, 0.88) | 0.08(0.07, 0.73) | 0.23(0.21, 2.07) |
| **Forest** | **10.27** | **1.76(0.62, 9.85)** | **2.81(1.14, 17.28)** | **3.18(1.31, 16.32)** | **7.75(3.07, 43.45)** |
| Grassland | 9.22 | 1.23(0.13, 6.20) | 2.84(0.30, 12.57) | 2.27(0.37, 10.06) | 6.34(0.80, 28.83) |
| Cropland | 9.19 | 1.21(0.16, 5.55) | 2.43(0.42, 11.10) | 2.71(0.43, 12.70) | 6.35(1.01, 29.35) |
| Others | 6.54 | 0.90(0.19, 7.28) | 1.67(0.39, 12.66) | 2.01(0.45, 15.05) | 4.58(1.03, 34.99) |
| **Subtotal** | **35.22** | **5.10(1.10, 28.88)** | **9.75(2.25, 53.61)** | **10.17(2.56, 54.13)** | **25.02(5.91, 136.62)** |
| **NO_y_** |  |  |  |  |  |
| Deciduous broadleaf forest | 2.44 | 0.60(0.29, 2.85) | 0.60(0.26, 3.24) | 0.58(0.29, 2.85) | 1.78(0.84, 8.94) |
| Evergreen broadleaf forest | 2.32 | 0.65(0.27, 2.39) | 0.37(0.16, 1.59) | 0.64(0.27, 2.39) | 1.66(0.70, 6.37) |
| Deciduous coniferous forest | 0.75 | 0.20(0.12, 0.58) | 0.18(0.11, 0.52) | 0.21(0.12, 0.58) | 0.59(0.35, 1.68) |
| Evergreen coniferous forest | 1.74 | 0.43(0.17, 2.44) | 0.39(0.15, 2.35) | 0.42(0.17, 2.44) | 1.24(0.49, 7.23) |
| Mixed forest | 0.31 | 0.08(0.07, 0.50) | 0.07(0.06, 0.47) | 0.08(0.07, 0.50) | 0.23(0.20, 1.47) |
| **Forest** | **7.56** | **1.96(0.85, 8.63)** | **1.61(0.74, 8.17)** | **1.93(0.93, 8.77)** | **5.50(2.52, 25.57)** |
| Grassland | 4.36 | 0.86(0.26, 4.61) | 1.07(0.29, 5.45) | 1.00(0.26, 4.61) | 2.93(0.81, 14.67) |
| Cropland | 4.29 | 1.01(0.36, 5.21) | 0.93(0.36, 5.04) | 1.04(0.36, 5.21) | 2.98(1.08, 15.46) |
| Others | 3.84 | 0.89(0.31, 6.26) | 0.91(0.32, 5.61) | 0.92(0.31, 6.26) | 2.72(0.94, 18.13) |
| **Subtotal** | **20.05** | **4.72(1.68, 23.52)** | **4.52(1.71, 24.27)** | **4.89(1.86, 24.84)** | **14.13(5.25, 72.63)** |
| **Total (NH_x_ + NO_y_)** | **55.27** | **9.82(2.78, 52.4)** | **14.27(3.96, 77.88)** | **15.06(4.42, 78.97)** | **39.15(11.16, 139.25)** |
| **NH_x_NO_y_** |  |  |  |  |  |
| Deciduous broadleaf forest | 5.64 | 1.79(1.01, 8.47) | 1.79(0.67, 9.59) | 1.09(0.36, 5.78) | 4.67(2.04, 23.84) |
| Evergreen broadleaf forest | 5.94 | 1.99(0.92, 8.21) | 1.96(0.73, 8.97) | 1.37(0.47, 6.32) | 5.32(2.12, 23.50) |
| Deciduous coniferous forest | 1.89 | 1.07(0.50, 3.15) | 0.44(0.18, 1.50) | 0.23(0.13, 0.71) | 1.74(0.81, 5.36) |
| Evergreen coniferous forest | 3.76 | 1.59(0.76, 9.00) | 1.11(0.29, 9.28) | 0.58(0.16, 4.85) | 3.28(1.21, 23.13) |
| Mixed forest | 0.59 | 0.26(0.25, 1.60) | 0.18(0.14, 1.58) | 0.08(0.06, 0.70) | 0.52(0.39, 3.88) |
| **Forest** | **17.81** | **6.70(3.45, 30.43)** | **5.48(2.01, 30.91)** | **3.35(1.17, 18.36)** | **15.53(5.46, 79.70)** |
| Grassland | 13.58 | 3.59(0.73, 16.52) | 3.51(0.42, 16.14) | 2.66(0.39, 11.62) | 9.76(1.15, 44.28) |
| Cropland | 13.49 | 3.28(1.00, 13.26) | 3.73(0.70, 17.64) | 3.31(0.69, 15.52) | 10.32(1.70, 46.42) |
| Others | 10.38 | 3.09(1.08, 20.71) | 2.83(0.52, 22.05) | 2.37(0.41, 16.49) | 8.29(1.60, 59.25) |
| **Subtotal** | **55.27** | **16.66(6.26, 80.91)** | **15.55(3.66, 86.74)** | **11.69(2.66, 61.99)** | **43.90(9.92, 229.64)** |

**Table S6** Mean retention of NH_x_ and NO_y_ in foliage, fine root, bark, stem, coarse root and branch of global forest trees. The numbers in brackets represent 95% confidence intervals.

| Ecosystem type | N retention (Tg N yr^-1^) | | | | | |
| --- | --- | --- | --- | --- | --- | --- |
|  | Foliage | Fine root | Bark | Stem | Coarse root | Branch |
| **NH_x_** | | | | | | |
| Deciduous broadleaf forest | 0.1273(0.0614, 1.0342) | 0.1673(0.0614, 1.0342) | 0.0269(0.0089, 0.1721) | 0.0989(0.0398, 0.5429) | 0.0631(0.0224, 0.3637) | 0.0974(0.0609, 0.6307) |
| Evergreen broadleaf forest | 0.0881(0.0341, 0.6029) | 0.1181(0.0341, 0.6029) | 0.0284(0.0089, 0.1563) | 0.1059(0.0409, 0.5915) | 0.0913(0.0277, 0.4046) | 0.1175(0.0720, 0.5390) |
| Deciduous coniferous forest | 0.0964(0.0165, 0.3264) | 0.0964(0.0165, 0.3264) | 0.0121(0.0046, 0.0379) | 0.0429(0.0166, 0.1220) | 0.0160(0.0063, 0.0518) | 0.0857(0.0506, 0.3072) |
| Evergreen coniferous forest | 0.1480(0.0416, 0.9325) | 0.1080(0.0416, 0.9325) | 0.0002(0.0001, 0.1666) | 0.0698(0.0226, 0.4879) | 0.0343(0.0089, 0.3387) | 0.0810(0.0513, 0.5088) |
| Mixed forest | 0.0242(0.0126, 0.2395) | 0.0242(0.0126, 0.2395) | 0.0028(0.0025, 0.0265) | 0.0085(0.0073, 0.0809) | 0.0044(0.0036, 0.0514) | 0.0155(0.0166, 0.1218) |
| **Forest** | **0.4839(0.1663,** **3.1355)** | **0.5139(0.1663,** **3.1355)** | **0.0704(0.0324,** **0.5594)** | **0.3260(0.1273,** **1.8252)** | **0.2090(0.0689,** **1.2102)** | **0.3972(0.2514,** **2.1074)** |
| **NO_y_** | | | | | | |
| Deciduous broadleaf forest | 0.1185(0.0557, 0.6676) | 0.1310(0.0700, 0.7855) | 0.1665(0.0729, 0.6140) | 0.0580(0.0322, 0.3274) | 0.0837(0.0606, 0.3861) | 0.0966(0.0488, 0.5256) |
| Evergreen broadleaf forest | 0.0870(0.0402, 0.3455) | 0.1048(0.0542, 0.7372) | 0.2540(0.1120, 0.6170) | 0.0374(0.0184, 0.1536) | 0.0669(0.0416, 0.2037) | 0.0969(0.0445, 0.3704) |
| Deciduous coniferous forest | 0.0425(0.0204, 0.1311) | 0.0361(0.0195, 0.1231) | 0.0658(0.0386, 0.1957) | 0.0283(0.0140, 0.0843) | 0.0482(0.0236, 0.1425) | 0.0390(0.0222, 0.1181) |
| Evergreen coniferous forest | 0.0967(0.0393, 0.5535) | 0.0704(0.0346, 0.5243) | 0.1107(0.0470, 0.4988) | 0.0569(0.0258, 0.2888) | 0.0856(0.0384, 0.3474) | 0.0762(0.0228, 0.4486) |
| Mixed forest | 0.0167(0.0140, 0.1171) | 0.0166(0.0135, 0.1008) | 0.0203(0.0189, 0.1303) | 0.0101(0.0089, 0.0642) | 0.0150(0.0148, 0.0823) | 0.0141(0.0106, 0.1050) |
| **Forest** | **0.3613(0.1695,** **1.8149)** | **0.3590(0.1918,** **2.2711)** | **0.6173(0.2893,** **2.0557)** | **0.1907(0.0992,** **0.9182)** | **0.2994(0.1790,** **1.1620)** | **0.3229(0.1489,** **1.5678)** |
| **Total (NH_x_ + NO_y_)** | **0.8453(0.3358,** **4.9504)** | **0.8730(0.3581,** **5.4066)** | **0.6877(0.3217,** **2.6152)** | **0.5167(0.2266,** **2.7435)** | **0.5084(0.2478,** **2.3722)** | **0.7200(0.4003,** **3.6752)** |

**Table S7** Utilizing plant and organic soil C/N ratio to estimate C sink caused by N deposition in global terrestrial ecosystems. The C/N ratio of plant and organic soil were obtained from different literature.

| Ecosystem type | C:N | | | | | | | | |
| --- | --- | --- | --- | --- | --- | --- | --- | --- | --- |
|  | foliage | branch | bark | Stem | coarse root | fine root | plant | litter | organic soil |
| Deciduous broadleaf forest | 30(29, 37) | \ | 131*(119, 144) | 320(317, 353) | 175 | 65(62, 69) |  | 65(64.93, 65.12) | 18(17, 19) |
| Evergreen broadleaf forest | 49(34, 55) | 56*(44, 59) | 59*(47, 67) | 407(340, 479) | 250(207, 300) | 64(53, 71) |  | 64(52, 71) | 19(17, 22) |
| Deciduous coniferous forest | 26(22, 27) | \ | \ | 190(136, 267) | 150 | 86(83, 93) |  | 87 | 20(16, 25) |
| Evergreen coniferous forest | 53(51, 57) | 62*(59, 70) | 139*(118, 190) | 454(436, 462) | 250(219, 276) | 92(82, 97) |  | 88(82, 93) | 21(20.72, 22.87) |
| Mixed forest | 28(27.56, 30.29) | \ | \ | 175(146, 236) | 41(37, 46) | 41(36, 44) |  | 49*(48.53, 51.74) | 13*(12.90, 14.44) |
| Grassland |  |  |  |  |  |  | 51(42, 63) | 67(54, 73) | 13(12.40,14.34) |
| Cropland |  |  |  |  |  |  | 62 | \ | 24 |
| Desert |  |  |  |  |  |  | 38 | \ | 27 |
| Tundra |  |  |  |  |  |  | 38 | 84(76, 92) | 25 |
| Wetland |  |  |  |  |  |  | 36*(35.26, 36.61) | \ | 22* |

* The C/N ratio was replaced by the mean value of each biome in the collected articles (1–5).

**Table S8** Comparison of the performance of different models for predicting N-induced C sink. Four linear regression models and four nonlinear models were applied. *R^2^* and root mean square error (RMSE) were used to evaluate the performance of the models.

| **Csink** | NH_x_ deposition | | NO_y_ deposition | |
| --- | --- | --- | --- | --- |
| Model | *R^2^* | RMSE | *R^2^* | RMSE |
| Linear regression | 0.33 | 6.78 | 0.50 | 4.95 |
| Multiple stepwise regression | 0.29 | 6.99 | 0.51 | 4.91 |
| Least angle regression | 0.29 | 7.11 | 0.49 | 5.02 |
| Elastic net model | 0.30 | 7.09 | 0.49 | 5.02 |
| Cubist | 0.63 | 5.06 | 0.65 | 4.19 |
| Boosted tree | 0.63 | 5.13 | 0.65 | 4.13 |
| Bagged tree | 0.62 | 5.16 | 0.57 | 4.59 |
| Random forest | 0.65 | 4.88 | 0.66 | 4.10 |

**Table S9** N-induced C sink in plant, organic soil and mineral soil of each biome on the globe. The numbers in brackets represent 95% confidence intervals.

| Ecosystem type | Area  (million km^2^) | C sink (Pg C yr^-1^) | | |
| --- | --- | --- | --- | --- |
|  |  | Plant | Organic layer | Mineral layer |
| **NH_x_** | | | | |
| Deciduous broadleaf forest | 12.12 | 0.0124(0.0072, 0.0622) | 0.0113(0.0071, 0.0388) | 0.0121(0.0086, 0.0284) |
| Evergreen broadleaf forest | 12.38 | 0.0096(0.0048, 0.0398) | 0.0101(0.0051, 0.0322) | 0.0202(0.0144, 0.0291) |
| Deciduous coniferous forest | 10.13 | 0.0903(0.0307, 0.1033) | 0.0274(0.0131, 0.0441) | 0.0161(0.0102, 0.0261) |
| Evergreen coniferous forest | 14.50 | 0.0329(0.0090, 0.1223) | 0.0149(0.0089, 0.0531) | 0.0139(0.0075, 0.0378) |
| Mixed forest | 3.41 | 0.0032(0.0024, 0.0210) | 0.0019(0.0010, 0.0099) | 0.0016(0.0006, 0.0074) |
| **Forest** | **52.54** | **0.1484(0.0541,** **0.3486)** | **0.0656(0.03552,** **0.1780)** | **0.0639(0.0413,** **0.1289)** |
| Grassland | 26.47 | 0.0270(0.0148, 0.2435) | 0.0310(0.0117, 0.0952) | 0.0280(0.0197, 0.0838) |
| Cropland | 12.23 | 0.0084(0.0077, 0.0531) | 0.0167(0.0069, 0.0505) | 0.0176(0.0096, 0.0394) |
| Others | 37.44 | 0.0326(0.0204, 0.3793) | 0.0400(0.0193, 0.1522) | 0.0399(0.0271, 0.1306 |
| **Subtotal** |  | **0.2164(0.0970,** **1.0244)** | **0.1532(0.0732,** **0.4759)** | **0.1493(0.0976,** **0.3827)** |
| **NO_y_** | | | | |
| Deciduous broadleaf forest | 12.12 | 0.0104(0.0054, 0.0452) | 0.0092(0.0053, 0.0216) | 0.0096(0.0069, 0.0191) |
| Evergreen broadleaf forest | 12.38 | 0.0085(0.0066, 0.0156) | 0.0109(0.0059, 0.0174) | 0.0145(0.0092, 0.0205) |
| Deciduous coniferous forest | 10.13 | 0.0401(0.0243, 0.0536) | 0.0093(0.0062, 0.0127) | 0.0122(0.0083, 0.0161) |
| Evergreen coniferous forest | 14.50 | 0.0254(0.0074, 0.0588) | 0.0088(0.0059, 0.0189) | 0.0100(0.0063, 0.0222) |
| Mixed forest | 3.41 | 0.0037(0.0023, 0.0143) | 0.0012(0.0004, 0.0042) | 0.0012(0.0006, 0.0048) |
| **Forest** | **52.54** | **0.0881(0.0459,** **0.1876)** | **0.0394(0.0237,** **0.0747)** | **0.0475(0.0312,** **0.0826)** |
| Grassland | 26.47 | 0.0215(0.0067, 0.1336) | 0.0179(0.0082, 0.0411) | 0.0208(0.0128, 0.0492) |
| Cropland | 12.23 | 0.0091(0.0026, 0.0438) | 0.0100(0.0038, 0.0252) | 0.0111(0.0064, 0.0261) |
| Others | 37.44 | 0.0439(0.0145, 0.1834) | 0.0235(0.0128, 0.0538) | 0.0276(0.0179, 0.0674) |
| **Subtotal** |  | **0.1626(0.0698,** **0.5484)** | **0.0907(0.0485,** **0.1949)** | **0.1070(0.0683,** **0.2253)** |
| **Total (NH_x_+NO_y_)** | **128.68** | **0.3790(0.1668,** **1.5728)** | **0.2439(0.1236,** **0.6708)** | **0.2563(0.1679,** **0.6080)** |

**Table S10** N-induced C sink in organic and mineral soils of each biome. Sensitivity tests were performed by using low C: N ratio in organic and mineral soils to compare with standard runs with central parameter values.

| Ecosystem type | Organic soil C:N ratio | | Mineral soil C:N ratio | | Organic soil C sink (Pg C yr^-1^) | | Mineral soil C sink (Pg C yr^-1^) | |  |
| --- | --- | --- | --- | --- | --- | --- | --- | --- | --- |
|  | Low C:N ratio | Standard run | Low C:N ratio | Standard run | Low C sink | Standard run | Low C sink | Standard run |  |
| Deciduous broadleaf forest | 20.11 | 21.03 | 17.12 | 18.30 | 0.0196 | 0.0205 | 0.0203 | 0.0217 |  |
| Evergreen broadleaf forest | 24.49 | 27.14 | 11.82 | 12.67 | 0.0189 | 0.0210 | 0.0324 | 0.0347 |  |
| Deciduous coniferous forest | 13.72 | 17.68 | 8.04 | 11.23 | 0.0285 | 0.0367 | 0.0203 | 0.0283 |  |
| Evergreen coniferous forest | 22.11 | 22.96 | 16.60 | 17.44 | 0.0228 | 0.0237 | 0.0227 | 0.0239 |  |
| Mixed forest | 12.90 | 13.67 | 13.04 | 13.40 | 0.0029 | 0.0031 | 0.0027 | 0.0028 |  |
| **Forest** | **18.67** | **20.50** | **13.32** | **14.61** | **0.0956** | **0.1050** | **0.1016** | **0.1114** |  |
| Grassland | 14.35 | 15.29 | 11.42 | 11.76 | 0.0459 | 0.0489 | 0.0474 | 0.0488 |  |
| Cropland | \ | \ | 11.00 | 11.10 | \ | 0.0267 | 0.0284 | 0.0287 |  |
| Others | \ | 21.05 | 11.48 | 12.13 | \ | 0.0635 | 0.0639 | 0.0675 |  |
| **Total** | **18.05** | **19.91** | **12.65** | **13.63** | **0.2211** | **0.2439** | **0.2379** | **0.2563** |  |

**Table S11** Carbon gain per unit N deposited was calculated by diving the estimated C sink by N deposition. The numbers in brackets represent 95% confidence intervals.

| Ecosystem type | Carbon gain per unit N deposition (kg C kg^-1^ N) | | | |
| --- | --- | --- | --- | --- |
|  | Plant | Organic soil | Mineral soil | Ecosystem |
| **NH_x_** | | | | |
| Deciduous broadleaf forest | 3.88(2.25, 19.43) | 3.53(2.20, 12.14) | 3.78(2.70, 8.86) | 11.19(9.45, 30.66) |
| Evergreen broadleaf forest | 2.65(1.32, 10.99) | 2.79(1.40, 8.88) | 5.58(3.97, 8.05) | 11.02(9.44, 25.32) |
| Deciduous coniferous forest | 79.21(26.96, 90.62) | 24.04(11.53, 38.68) | 14.12(8.91, 22.92) | 117.37(34.11, 135.88) |
| Evergreen coniferous forest | 16.29(4.47, 60.54) | 7.38(4.40, 26.27) | 6.88(3.70, 18.72) | 30.54(14.81, 97.97) |
| Mixed forest | 11.03(8.27, 72.51) | 6.55(3.62, 34.16) | 5.52(2.20, 25.68) | 23.10(200.91, 121.76) |
| **Forest** | **14.45(5.27, 33.94)** | **6.39(3.43, 17.34)** | **6.22(4.02, 12.55)** | **27.06(13.56, 56.27)** |
| Grassland | 2.93(1.60, 26.41) | 3.36(1.27, 10.32) | 3.04(2.13, 9.09) | 9.33(5.08, 40.55) |
| Cropland | 0.91(0.83, 5.78) | 1.82(0.75, 5.50) | 1.92(1.04, 4.29) | 4.65(2.91, 12.14) |
| Others | 4.98(3.12, 58.00) | 6.12(2.96, 23.27) | 6.10(4.14, 19.97) | 17.20(11.32, 113.29) |
| **Subtotal** | **6.14(2.75, 29.09)** | **4.35(2.08, 13.51)** | **4.24(2.77, 10.87)** | **14.73(8.15, 51.23)** |
| **NO_y_** | | | | |
| Deciduous broadleaf forest | 4.26(1.68, 14.13) | 3.77(1.65, 6.74) | 3.93(2.14, 5.97) | 11.97(5.91, 29.74) |
| Evergreen broadleaf forest | 3.66(1.82, 4.31) | 4.70(1.62, 4.81) | 6.25(2.55, 5.67) | 14.61(8.28, 20.83) |
| Deciduous coniferous forest | 53.47(21.31, 47.00) | 12.40(5.42, 11.10) | 16.27(7.28, 14.09) | 82.13(47.17, 81.98) |
| Evergreen coniferous forest | 14.60(3.65, 29.13) | 5.06(2.94, 9.37) | 5.75(3.10, 10.98) | 25.40(13.47, 48.47) |
| Mixed forest | 11.94(8.02, 49.39) | 3.87(1.36, 14.47) | 3.87(1.98, 16.49) | 19.68(16.47, 60.98) |
| **Forest** | **11.65(4.47, 18.26)** | **5.21(2.30, 7.28)** | **6.28(3.04, 8.05)** | **23.15(12.90, 37.78)** |
| Grassland | 4.93(0.73, 14.49) | 4.11(0.89, 4.46) | 4.77(1.39, 5.33) | 13.81(5.25, 38.88) |
| Cropland | 2.12(0.28, 4.76) | 2.33(0.41, 2.74) | 2.59(0.70, 2.83) | 7.04(2.91, 17.32) |
| Others | 11.43(2.22, 28.05) | 6.12(1.96, 8.23) | 7.19(2.73, 10.31) | 24.74(8.61, 61.85) |
| **Subtotal** | **8.11(1.98, 15.57)** | **4.52(1.38, 5.53)** | **5.34(1.94, 6.40)** | **17.97(8.28, 38.25)** |
| **Total (NH_x_+NO_y_)** | **6.86(1.30, 12.22)** | **4.41(0.96, 5.21)** | **4.64(1.30, 4.72)** | **15.91(3.52, 19.98)** |

**Table S12** The estimation of global terrestrial ecosystem total N deposition and its contribution to C sink, as well as the carbon gain per unit N deposition of global terrestrial ecosystems by stoichiometric scale, meta-analysis and model analysis.

| Method used to estimate C sink | Ecosystem | Total area (million km^2^) | N deposition  (Tg N yr^-1^) | Period**^a^** | C sink  (Pg C yr^-1^) | C gain per unit N deposited  (kg C kg^-1^ N) | | Reference |
| --- | --- | --- | --- | --- | --- | --- | --- | --- |
| Stoichiometric scaling | Forest | 52.54 | 17.83 | 2010-2014 | 0.4529(0.2368, 0.8635) | 25.40(13.38, 48.43) | | This study |
|  | Grassland | 26.47 | 13.58 |  | 0.1460(0.0698, 0.5433) | 10.75(5.14, 40.01) | |  |
|  | Cropland | 12.23 | 13.49 |  | 0.0728(0.0393, 0.1859) | 5.40(2.91, 13.78) | |  |
|  | Others | 37.44 | 10.38 |  | 0.2075(0.1071, 0.9784) | 19.99(10.32, 94.26) | |  |
| Stoichiometric scaling | Forest | 32.60 | 15.26 | 2009 | $\text{0.352}_{\text{0.276}}^{\text{0.448}}$ | 23.07 | | De Vries et al., 2014(6) |
| Stoichiometric scaling | Forest | 42.00 | 24.90 | 2010 | $\text{0.720}_{\text{0.490}}^{\text{0.960}}$ | 28.92 | | Gurmesa et al., 2022(7) |
| Stoichiometric scaling | Forest | 40.60 | 21.00 | 2001 | $\text{0.250}_{\text{0.050}}^{\text{0.890}}$ | 11.90 | | Du and de Vries, 2018(8) |
| Stoichiometric scaling | Forest |  | 5.10 | 1990s | 0.251 | 49.22 | | Nadelhoffer et al., 1999(9) |
| Stoichiometric scaling | Forest | 25.87 | 16.25 | 2010 | 0.376 | 23.14 | | Wang et al., 2017(4) |
| Stoichiometric scaling | Forest | 48.50 | 5.50 | 1985 | 0.091 | 16.55 | | Peterson and Melillo, 1985(10) |
|  | Shrubland | 8.50 | 0.50 | 1985 | 0.007 | 14.00 | |  |
| Meta-analysis **^b^** | Forest | 40.20 | 21.71 |  | $\text{0.177}_{\text{0.112}}^{\text{0.243}}$ | 8.15 | | Schulte-Uebbing and De Vries, 2018(11) |
| Meta-analysis**^c^** | Forest | 57.22 |  |  | 0.350 |  | | Deng et al., 2019(12) |
|  | Grassland | 38.72 |  |  | 0.150 |  | |  |
|  | Cropland | 23.82 |  |  | 1.210 |  | |  |
|  | Wetland | 20.90 |  |  | 0.020 |  | |  |
| Meta-analysis**^d^** | Forest |  |  |  | 0.321 |  | | Xiao et al., 2023(13) |
|  | Grassland |  |  |  | 0.186 |  | |  |
| DGVM | Forest | 38.51 | 20.30 | 1900-2007 | $\text{0.460}_{\text{0.180}}^{\text{0.740}}$ | 22.66 | | Fleischer et al., 2015(14) |
| DGVM | Terrestrial |  |  | 1990s | 0.260 |  | | Jain et al., 2009(15) |
| CLIMBER-2 with BIOME-BGC | Terrestrial |  |  | 1990s | 1.020 |  | | Churkina et al., 2009(16) |
| CLM4.5-BGC | Terrestrial |  |  | 2010-2016 | 0.690 | |  | O’Sullivan et al., 2019(17) |

a: indicated the N deposition period.

b: N-induced forest C sink include only aboveground and belowground woody biomass.

c: N-induced each biome C sink only in soil layer.

d: N-induced each biome C sink only in organic soil layer.

**References:**

1. V. Haverd, *et al.*, A new version of the CABLE land surface model (subversion revision r4601) incorporating land use and land cover change, woody vegetation demography, and a novel optimisation-based approach to plant coordination of photosynthesis. *Geosci. Model Dev.* **11**, 2995–3026 (2018).

2. S. Zechmeister-Boltenstern, *et al.*, The application of ecological stoichiometry to plant–microbial–soil organic matter transformations. *Ecol. Monogr.* **85**, 133–155 (2015).

3. C. C. Cleveland, *et al.*, Patterns of new versus recycled primary production in the terrestrial biosphere. *Proc Natl Acad Sci U S A* **110**, 12733–12737 (2013).

4. R. Wang, *et al.*, Global forest carbon uptake due to nitrogen and phosphorus deposition from 1850 to 2100. *Global Change Biology* **23**, 4854–4872 (2017).

5. Y. Wang, *et al.*, GOLUM-CNP v1.0: a data-driven modeling of carbon, nitrogen and phosphorus cycles in major terrestrial biomes. *Geosci. Model Dev.* **11**, 3903–3928 (2018).

6. W. de Vries, E. Du, K. Butterbach-Bahl, Short and long-term impacts of nitrogen deposition on carbon sequestration by forest ecosystems. *Current Opinion in Environmental Sustainability* **9–10**, 90–104 (2014).

7. G. A. Gurmesa, *et al.*, Retention of deposited ammonium and nitrate and its impact on the global forest carbon sink. *Nat Commun* **13**, 880 (2022).

8. E. Du, W. de Vries, Nitrogen-induced new net primary production and carbon sequestration in global forests. *Environmental Pollution* **242**, 1476–1487 (2018).

9. K. J. Nadelhoffer, *et al.*, Nitrogen deposition makes a minor contribution to carbon sequestration in temperate forests. **398** (1999).

10. B. J. Peterson, J. M. Melillo, The potential storage of carbon caused by eutrophication of the biosphere. *Tellus B* **37B**, 117–127 (1985).

11. L. Schulte-Uebbing, W. de Vries, Global-scale impacts of nitrogen deposition on tree carbon sequestration in tropical, temperate, and boreal forests: A meta-analysis. *Glob. Change Biol.* **24**, E416–E431 (2018).

12. L. Deng, *et al.*, Soil GHG fluxes are altered by N deposition: New data indicate lower N stimulation of the N2O flux and greater stimulation of the calculated C pools. *Global Change Biology* **26**, 2613–2629 (2020).

13. S. Xiao, *et al.*, Enhanced CO2 uptake is marginally offset by altered fluxes of non-CO2 greenhouse gases in global forests and grasslands under N deposition. *Glob Chang Biol* **29**, 5829–5849 (2023).

14. K. Fleischer, *et al.*, Low historical nitrogen deposition effect on carbon sequestration in the boreal zone. *J. Geophys. Res.: Biogeosci.* **120**, 2542–2561 (2015).

15. A. Jain, *et al.*, Nitrogen attenuation of terrestrial carbon cycle response to global environmental factors. *Global Biogeochemical Cycles* **23** (2009).

16. G. Churkina, *et al.*, Synergy of rising nitrogen depositions and atmospheric CO2 on land carbon uptake moderately offsets global warming. *Global Biogeochemical Cycles* **23** (2009).

17. M. O’Sullivan, *et al.*, Have Synergies Between Nitrogen Deposition and Atmospheric CO2 Driven the Recent Enhancement of the Terrestrial Carbon Sink? *Global Biogeochemical Cycles* **33**, 163–180 (2019).

**References**. List of all the references used in this study.

1. K. M. Andersen, J. R. Mayor, B. L. Turner, Plasticity in nitrogen uptake among plant species with contrasting nutrient acquisition strategies in a tropical forest. *Ecology* **98**, 1388–1398 (2017).

2. L. C. Andresen, S. Jonasson, L. Ström, A. Michelsen, Uptake of pulse injected nitrogen by soil microbes and mycorrhizal and non-mycorrhizal plants in a species-diverse subarctic heath ecosystem. *Plant Soil* **313**, 283–295 (2008).

3. L. C. Andresen, A. Michelsen, Off-season uptake of nitrogen in temperate heath vegetation. *Oecologia* **144**, 585–597 (2005).

4. A. Bähring, A. Fichtner, U. Friedrich, G. von Oheimb, W. Härdtle, Bryophytes and organic layers control uptake of airborne nitrogen in low-N environments. *Front. Plant Sci.* **8** (2017).

5. J. E. Barrett, I. C. Burke, Nitrogen retention in semiarid ecosystems across a soil organic-matter gradient. *Ecol. Appl.* **12**, 878 (2002).

6. A. Bird, S. A. Watmough, M. A. Carson, N. Basiliko, A. McDonough, Nitrogen retention of terricolous lichens in a northern alberta jack pine forest. *Ecosystems* **22**, 1308–1324 (2019).

7. E. Björkman, G. Lundeberg, H. Nömmik, Distribution and balance of N15 labelled fertilizer nitrogen applied to young pine trees (pinus silvestris L.). *Stud. for. suec.* (1967).

8. D. Bryan Dail, *et al.*, Distribution of nitrogen-15 tracers applied to the canopy of a mature spruce-hemlock stand, howland, maine, USA. *Oecologia* **160**, 589–599 (2009).

9. N. Buchmann, G. Gebauer, E.-D. Schulze, Partitioning of 15N-labeled ammonium and nitrate among soil, litter, below- and above-ground biomass of trees and understory in a 15-year-old picea abies plantation. *Biogeochemistry* **33** (1996).

10. J. Calvo-Fernández, E. Marcos, L. Calvo, W. Härdtle, Allocation patterns of airborne nitrogen in mountainous heathlands – a 15 N tracer study in the cantabrian mountains (NW spain). *Ecol. Eng.* **84**, 128–135 (2015).

11. S. X. Chang, G. F. Weetman, C. M. Preston, K. McCullough, J. Barker, Effect of understory competition on distribution and recovery of 15N applied to a western red cedar–western hemlock clear-cut site. *Can. J. For. Res.* **26**, 313–321 (1996).

12. W.-J. Choi, S. X. Chang, X. Hao, Soil retention, tree uptake, and tree resorption of15NH4NO3and NH415NO3applied to trembling and hybrid aspens at planting. *Can. J. For. Res.* **35**, 823–831 (2005).

13. S. Choudhary, A. Blaud, A. M. Osborn, M. C. Press, G. K. Phoenix, Nitrogen accumulation and partitioning in a high arctic tundra ecosystem from extreme atmospheric N deposition events. *Science of The Total Environment* **554–555**, 303–310 (2016).

14. L. M. Christenson, G. M. Lovett, M. J. Mitchell, P. M. Groffman, The fate of nitrogen in gypsy moth frass deposited to an oak forest floor. *Oecologia* **131**, 444–452 (2002).

15. F. E. Clark, Internal cycling of nitrogen in shortgrass prairie. *Ecology* **58**, 1322–1333 (1977).

16. P. W. Clinton, D. J. Mead, Competition for nitrogen between Pinusradiata and pasture. I. Recovery of 15N after one growing season. *Can. J. For. Res.* **24**, 882–888 (1994).

17. H. Crook, B. Emmett, P. Johnes, B. Reynolds, Carbon and nitrogen cycling in upland wetlands. (2002).

18. X. Cui, *et al.*, Impacts of water and nitrogen addition on nitrogen recovery in haloxylon ammodendron dominated desert ecosystems. *Science of The Total Environment* **601–602**, 1280–1288 (2017).

19. C. J. Curtis, *et al.*, Nitrogen saturation in UK moorlands: the critical role of bryophytes and lichens in determining retention of atmospheric N deposition. *J. Appl. Ecol.* **42**, 507–517 (2005).

20. M. A. Dawes, P. Schleppi, F. Hagedorn, The fate of nitrogen inputs in a warmer alpine treeline ecosystem: a 15N labelling study. *J. Ecol.* **105**, 1723–1737 (2017).

21. J. V. Dean, D. D. Biesboer, Loss and uptake of 15 N-ammonium in submerged soils of a cattail marsh. *Am. J. Bot.* **72**, 1197 (1985).

22. C. J. Dell, M. A. Williams, C. W. Rice, Partitioning of nitrogen over five growing seasons in tallgrass prairie. *Ecology* **86**, 1280–1287 (2005).

23. H. E. Epstein, I. C. Burke, A. R. Mosier, Plant effects on nitrogen retention in shortgrass steppe 2 years after 15N addition. *Oecologia* **128**, 422–430 (2001).

24. T. J. Fahey, *et al.*, Transport of carbon and nitrogen between litter and soil organic matter in a northern hardwood forest. *Ecosystems* **14**, 326–340 (2011).

25. S. Feigenbaum, A. Hadas, Utilization of fertilizer nitrogen‐nitrogen‐15 by field‐grown alfalfa. *Soil Sci. Soc. Am. J.* **44**, 1006–1010 (1980).

26. Z. Feng, R. Brumme, Y.-J. Xu, N. Lamersdorf, Tracing the fate of mineral N compounds under high ambient N deposition in a Norway spruce forest at solling/germany. *For. Ecol. Manage.* **255**, 2061–2073 (2008).

27. U. Friedrich, *et al.*, Fate of airborne nitrogen in heathland ecosystems: a 15N tracer study: FATE OF AIRBORNE NITROGEN IN HEATHLANDS. *Global Change Biol.* **17**, 1549–1559 (2010).

28. C. B. Fuss, *et al.*, Retention of nitrate-N in mineral soil organic matter in different forest age classes. *Ecosystems* **22**, 1280–1294 (2019).

29. C. T. Garten, D. J. Brice, D. E. Todd, Short‐term recovery of ammonium‐15Nitrogen applied to a temperate forest inceptisol and ultisol in east tennessee, USA. *Commun. Soil Sci. Plant Anal.* **38**, 2693–2704 (2007).

30. C. L. Goodale, Multiyear fate of a15N tracer in a mixed deciduous forest: retention, redistribution, and differences by mycorrhizal association. *Global Change Biol.* **23**, 867–880 (2016).

31. C. L. Goodale, *et al.*, Soil processes drive seasonal variation in retention of 15N tracers in a deciduous forest catchment. *Ecology* **96**, 2653–2668 (2015).

32. P. Gundersen, Effects of enhanced nitrogen deposition in a spruce forest at klosterhede, denmark, examined by moderate NH4NO3 addition. *For. Ecol. Manage.* **101**, 251–268 (1998).

33. G. A. Gurmesa, *et al.*, Retention of deposited ammonium and nitrate and its impact on the global forest carbon sink. *Nat Commun* **13**, 880 (2022).

34. S. C. Hart, M. K. Firestone, E. A. Paul, J. L. Smith, Flow and fate of soil nitrogen in an annual grassland and a young mixed-conifer forest. *Soil Biol. Biochem.* **25**, 431–442 (1993).

35. E.-L. S. Hinckley, B. A. Ebel, R. T. Barnes, S. F. Murphy, S. P. Anderson, Erratum to: critical zone properties control the fate of nitrogen during experimental rainfall in montane forests of the colorado front range. *Biogeochemistry* **134**, 371–371 (2017).

36. E. S. Hinckley, R. T. Barnes, S. P. Anderson, M. W. Williams, S. M. Bernasconi, Nitrogen retention and transport differ by hillslope aspect at the rain‐snow transition of the colorado front range. *J. Geophys. Res.: Biogeosci.* **119**, 1281–1296 (2014).

37. K. S. Hofmockel, *et al.*, Sources of increased N uptake in forest trees growing under elevated CO2: results of a large-scale 15N study. *Global Change Biol.* **17**, 3338–3350 (2011).

38. S. Li, *et al.*, Fate of atmospherically deposited <scp>NH</scp>4+ and <scp>NO</scp>3− in two temperate forests in China: temporal pattern and redistribution. *Ecol. Appl.* **29**, e01920 (2019).

39. K. J. Holland, “Fate of nitrogen in alpine tundra.”

40. S. M. Holub, K. Lajtha, The fate and retention of organic and inorganic 15N-nitrogen in an old-growth forest soil in western oregon. *Ecosystems* **7** (2004).

41. Z. Jia, *et al.*, Deepened snow loosens temporal coupling between plant and microbial N utilization and induces ecosystem N losses. *Global Change Biology* **28**, 4655–4667 (2022).

42. C. J. Koopmans, A. Tietema, A. W. Boxman, The fate of15N enriched throughfall in two coniferous forest stands at different nitrogen deposition levels. *Biogeochemistry* **34**, 19–44 (1996).

43. S. Lamontagne, S. L. Schiff, R. J. Elgood, Recovery of15N-labelled nitrate applied to a small upland boreal forest catchment. *Can. J. For. Res.* **30**, 1165–1177 (2000).

44. W. Liu, *et al.*, In situ 15N labeling experiment reveals different long‐term responses to ammonium and nitrate inputs in N‐saturated subtropical forest. *J. Geophys. Res.: Biogeosci.* **122**, 2251–2264 (2017).

45. R. Lutter, *et al.*, Belowground resource utilization in monocultures and mixtures of scots pine and Norway spruce. *For. Ecol. Manage.* **500**, 119647 (2021).

46. L. Ma, C. Zhang, Y. Lv, R. Wang, The retention dynamics of early-spring N input in a temperate forest ecosystem: implications for winter N deposition. *Global Ecol. Conserv.* **33**, e01966 (2022).

47. J. Mao, *et al.*, Unexpected high retention of15N‐labeled nitrogen in a tropical legume forest under long‐term nitrogen enrichment. *Global Change Biol.* **28**, 1529–1543 (2021).

48. V. Martinsen, G. Austrheim, A. Mysterud, J. Mulder, Effects of herbivory on N-cycling and distribution of added 15NH 4 + in N-limited low-alpine grasslands. *Plant Soil* **347**, 279–292 (2011).

49. M. Martí-Roura, P. Casals, J. Romanyà, Long-term retention of post-fire soil mineral nitrogen pools in Mediterranean shrubland and grassland. *Plant Soil* **371**, 521–531 (2013).

50. D. J. Mead, W. L. Pritchett, Fertilizer movement in a a slash pine ecosystem: II. N distribution after two growing seasons. *Plant Soil* **43**, 467–478 (1975).

51. J. Melin, H. Nômmik, U. Lohm, J. Flower-Ellis, Fertilizer nitrogen budget in a scots pine ecosystem attained by using root-isolated plots and15N tracer technique. *Plant Soil* **74**, 249–263 (1983).

52. P. Micks, M. R. Downs, A. H. Magill, K. J. Nadelhoffer, J. D. Aber, Decomposing litter as a sink for 15N<math><msup><mi></mi><mn>15</mn></msup><mtext>N</mtext></math>-enriched additions to an oak forest and a red pine plantation. *For. Ecol. Manage.* **196**, 71–87 (2004).

53. J. T. Moraghan, Loss and assimilation of 15N-nitrate added to a north dakota cattail marsh. *Aquat. Bot.* **46**, 225–234 (1993).

54. I. Morier, C. Guenat, R. Siegwolf, J. Védy, P. Schleppi, Dynamics of atmospheric nitrogen deposition in a temperate calcareous forest soil. *J. Environ. Qual.* **37**, 2012–2021 (2008).

55. I. Morier, P. Schleppi, M. Saurer, I. Providoli, C. Guenat, Retention and hydrolysable fraction of atmospherically deposited nitrogen in two contrasting forest soils in Switzerland. *Eur. J. Soil Sci.* **61**, 197–206 (2010).

56. K. A. Morris, R. K. F. Nair, G. Moreno, M. Schrumpf, M. Migliavacca, Fate of N additions in a multiple resource‐limited mediterranean oak savanna. *Ecosphere* **10** (2019).

57. K. J. Nadelhoffer, *et al.*, Nitrogen deposition makes a minor contribution to carbon sequestration in temperate forests. **398** (1999).

58. K. J. Nadelhoffer, B. P. Colman, W. S. Currie, A. Magill, J. D. Aber, Decadal-scale fates of tracers added to oak and pine stands under ambient and elevated N inputs at the harvard forest (USA). *For. Ecol. Manage.* **196**, 89–107 (2004).

59. K. Pan, Z. Xu, T. J. Blumfield, S. Tutua, M. Lu, Application of (15NH4)2SO4 to study N dynamics in hoop pine plantation and adjacent native forest of subtropical australia: the effects of injection depth and litter addition. *J. Soils Sediments* **9**, 515–525 (2009).

60. S. S. Perakis, L. O. Hedin, Fluxes and fates of nitrogen in soil of an unpolluted old-growth temperate forest, southern Chile. *Ecology* **82**, 2245 (2001).

61. C. M. Preston, V. G. Marshall, K. McCullough, D. J. Mead, Fate of 15N-labelled fertilizer applied on snow at two forest sites in british columbia. *Can. J. For. Res.* **20**, 1583–1592 (1990).

62. I. Providoli, H. Bugmann, R. Siegwolf, N. Buchmann, P. Schleppi, Flow of deposited inorganic N in two gleysol-dominated mountain catchments traced with 15NO3− and 15NH4+. *Biogeochemistry* **76**, 453–475 (2005).

63. I. Providoli, H. Bugmann, R. Siegwolf, N. Buchmann, P. Schleppi, Pathways and dynamics of 15NO3− and 15NH4+ applied in a mountain picea abies forest and in a nearby meadow in central switzerland. *Soil Biol. Biochem.* **38**, 1645–1657 (2006).

64. E. Remy, K. Wuyts, K. Verheyen, P. Gundersen, P. Boeckx, Altered microbial communities and nitrogen availability in temperate forest edges. *Soil Biol. Biochem.* **116**, 179–188 (2018).

65. J. P. Schimel, M. K. Firestone, Nitrogen incorporation and flow through a coniferous forest soil profile. *Soil Sci. Soc. Am. J.* **53**, 779–784 (1989).

66. M. Schlingmann, *et al.*, Intensive slurry management and climate change promote nitrogen mining from organic matter-rich montane grassland soils. *Plant Soil* **456**, 81–98 (2020).

67. B. Seely, K. Lajtha, Application of a 15 N tracer to simulate and track the fate of atmospherically deposited N in the coastal forests of the waquoit bay watershed, cape cod, massachusetts. *Oecologia* **112**, 393–402 (1997).

68. W. Sheng, *et al.*, Sinks for inorganic nitrogen deposition in forest ecosystems with low and high nitrogen deposition in China. *PLOS One* **9**, e89322 (2014).

69. C. W. Swanston, D. D. Myrold, Incorporation of nitrogen from decomposing red alder leaves into plants and soil of a recent clearcut in oregon. *Can. J. For. Res.* **27**, 1496–1502 (1997).

70. P. H. Templer, G. M. Lovett, K. C. Weathers, S. E. Findlay, T. E. Dawson, Influence of tree species on forest nitrogen retention in the catskill mountains, new york, USA. *Ecosystems* **8**, 1–16 (2005).

71. A. Tietema, B. A. Emmett, P. Gundersen, O. J. Kjønaas, C. J. Koopmans, The fate of 15N-labelled nitrogen deposition in coniferous forest ecosystems. *For. Ecol. Manage.* **101**, 19–27 (1998).

72. A. M. Tye, *et al.*, The fate of 15N added to high arctic tundra to mimic increased inputs of atmospheric nitrogen released from a melting snowpack. *Global Change Biol.* **11**, 1640–1654 (2005).

73. L. Veerman, *et al.*, The long-term fate of deposited nitrogen in temperate forest soils. *Biogeochemistry* **150**, 1–15 (2020).

74. Q. Wan, *et al.*, Different fates and retention of deposited NH4+ and NO3− in an alpine grassland in northwest China: a 15N tracer study. *Environ. Exp. Bot.* **201**, 104989 (2022).

75. A. Wang, *et al.*, Fates of atmospheric deposited nitrogen in an asian tropical primary forest. *For. Ecol. Manage.* **411**, 213–222 (2018).

76. A. Wang, *et al.*, Dynamics and multi‐annual fate of atmospherically deposited nitrogen in montane tropical forests. *Global Change Biol.* **27**, 2076–2087 (2021).

77. I. Watanabe, B. C. Padre, Inorganic nitrogen movement in tropical soils: II. The fate of ammonium fertilizer in well and poorly drained soils in the rainy season. *Soil Sci. Plant Nutr.* **25**, 627–635 (1979).

78. W. W. Wessel, A. W. Boxman, C. Cerli, E. E. van Loon, A. Tietema, Long-term stabilization of 15N-labeled experimental NH4+ deposition in a temperate forest under high N deposition. *Sci. Total Environ.* **768**, 144356 (2021).

79. D. Xie, *et al.*, Long‐term 15N balance after single‐dose input of 15N‐labeled NH4+ and NO3− in a subtropical forest under reducing N deposition. *Global Biogeochem. Cycles* **35** (2021).

80. L. Yang, *et al.*, Land-use change reduces soil nitrogen retention of both particulate and mineral-associated organic matter in a temperate grassland. *Catena* **216**, 106432 (2022).

81. L. Yu, R. Kang, J. Mulder, J. Zhu, P. Dörsch, Distinct fates of atmogenic NH4 + and NO3 − in subtropical, N-saturated forest soils. *Biogeochemistry* **133**, 279–294 (2017).

82. D. R. Zak, K. S. Pregitzer, W. E. Holmes, A. J. Burton, G. P. Zogg, Anthropogenic N deposition and the fate of 15NO3- in a northern hardwood ecosystem. *Biogeochemistry* **69**, 143–157 (2004).

83. D. R. Zak, W. E. Holmes, K. S. Pregitzer, Atmospheric co2and o3alter the flow of15n in developing forest ecosystems. *Ecology* **88**, 2630–2639 (2007).

84. M. Zistl-Schlingmann, S. Kwatcho Kengdo, R. Kiese, M. Dannenmann, Management intensity controls nitrogen-use-efficiency and flows in grasslands—a 15N tracing experiment. *Agronomy* **10**, 606 (2020).

85. G. P. Zogg, D. R. Zak, K. S. Pregitzer, A. J. Burton, Microbial immobilization and the retention of anthropogenic nitrate in a northern hardwood forest. *Ecology* **81**, 1858 (2000).
